# Investigating the functional components of YAP condensates

**DOI:** 10.1091/mbc.E25-11-0532

**Published:** 2026-05-13

**Authors:** Janelle S. Bellot, Siyuan Hao, Yihuan Li, Danfeng Cai

**Affiliations:** ^a^Department of Biochemistry and Molecular Biology, Johns Hopkins Bloomberg School of Public Health, Baltimore, Maryland 21205; ^b^Center for Cell Dynamics, Johns Hopkins University School of Medicine, Baltimore, Maryland 21205; ^c^Department of Biophysics and Biophysical Chemistry, Johns Hopkins School of Medicine, Baltimore, Maryland 21205; ^d^Department of Oncology, Johns Hopkins School of Medicine, Baltimore, Maryland 21205; Carnegie Mellon University

## Abstract

Yes-associated protein (YAP) condensates are critical for cell survival under hyperosmotic stress, yet how these condensates execute their specific functions remains incompletely understood. Here, we employed proximity-based proteomics to identify YAP-interacting proteins in both the diffuse and condensate-forming states. Upon YAP condensate formation, the composition of YAP-interacting proteins changed markedly. Moreover, YAP condensate components transitioned from an initial chromatin-clustering state to a subsequent transcriptional activation state. Using immunofluorescence, we verified that JUNB, TCF12, and IFI16 were enriched within endogenous YAP condensates. Notably, JUNB and TCF12 are also required for YAP condensate formation and function, as their depletion completely abolished condensate assembly and downstream gene expression. Together, these findings identify novel components essential for YAP condensate formation and illuminate their roles in the hyperosmotic stress response, providing a foundation for future therapeutic strategies targeting YAP condensates.

## Abstract (executive summary)

- YAP plays key roles in chromatin reorganization and transcriptional activation. However, the mechanisms by which YAP condensates exert these functions remain incompletely understood.
- We employed proximity-based proteomics to identify novel components of YAP condensates, such as JUNB and TCF12. These components are not only enriched within YAP condensates but are also required for their formation.
- We revealed how distinct signaling pathways converge at condensates to coordinate downstream functions. Given YAP's critical role in oncogenesis, this work also establishes a foundation for future efforts to therapeutically target YAP condensates in cancer and other diseases.

## INTRODUCTION

Yes-associated protein (YAP) is a master transcriptional coactivator that drives the expression of cell proliferation and survival-related genes. It is highly expressed during organismal development, and reactivated in adulthood during wound healing or in disease processes such as cancer (Pan, 2010; Meng *et al.*, 2016). YAP interacts with transcription factors (TFs), most prominently the TEA domain (TEAD) family TFs (Zhao *et al.*, 2008). Its nuclear translocation and activities are tightly controlled by the Hippo pathway, which contains the kinase cascade involving MST/LATS in the cytoplasm (Meng *et al.*, 2016; Zheng and Pan, 2019). In addition, YAP activities are regulated by various stress signals such as osmotic, ER, and hypoxic stresses (Ma *et al.*, 2015; Wu *et al.*, 2015; Ma *et al.*, 2016; Hong *et al.*, 2017; Moon *et al.*, 2017). However, the spatiotemporal organization of YAP activities during signal activation, especially after entering the nucleus, remains poorly understood.

**FIGURE 1:**
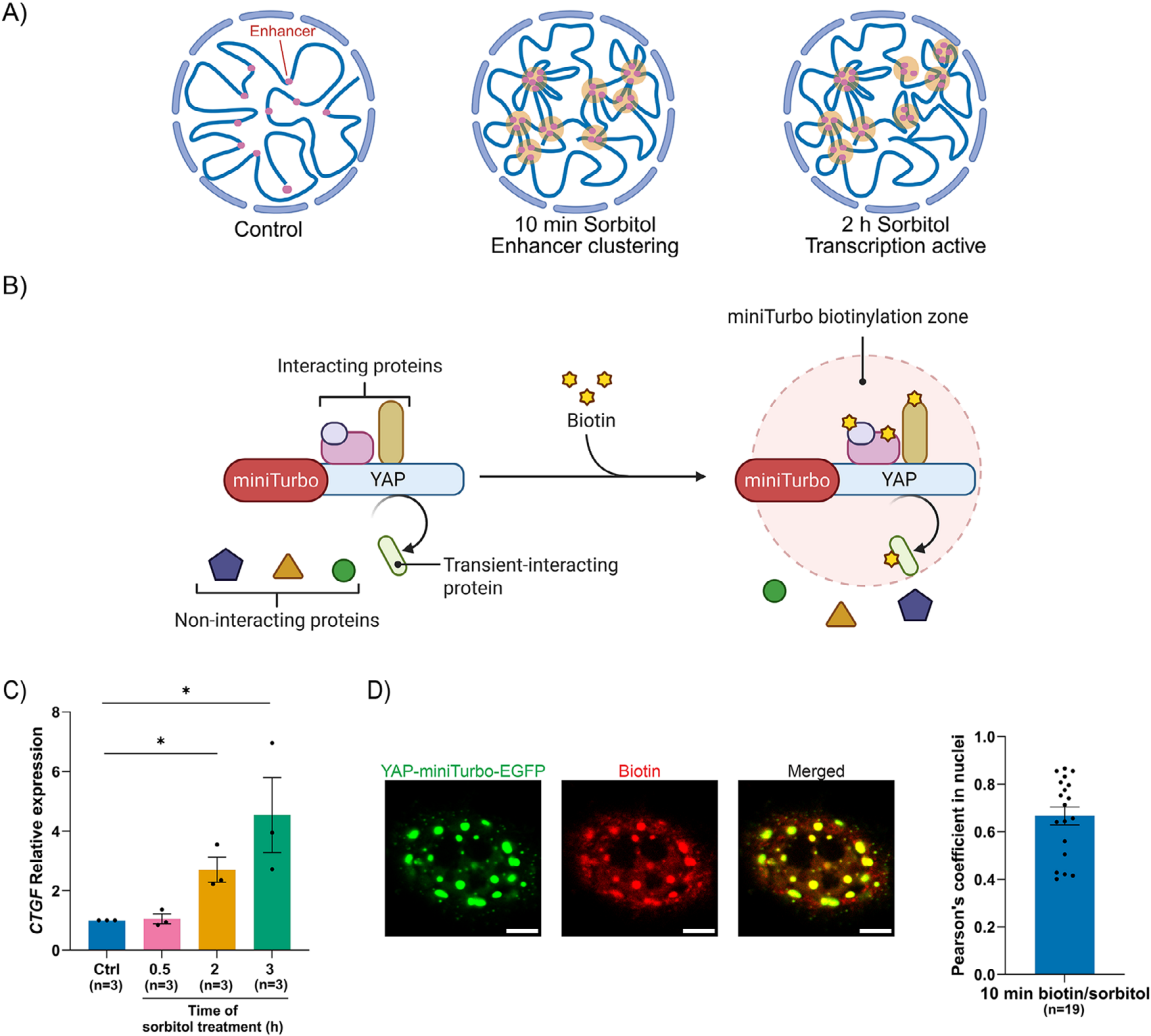
Proximity-based proteomics to identify YAP-interacting proteins. (A) Illustration of YAP condensate functions in enhancer clustering and transcription activation after 5 min and 2 h sorbitol treatment respectively, adapted from (Cai *et al.*, 2019). (B) Proximity labeling to identify components of YAP condensates. The miniTurbo-YAP plasmid is transfected into HeLa cells. Upon addition of biotin, YAP-proximal proteins are biotinylated (star). Conditions include 10 min and 2 h after hyperosmotic stress to assay for different components of YAP condensates. (C) Expression level of YAP target gene, *CTGF*, was quantified by qPCR from HeLa cell transfected with miniTurbo-YAP-EGFP after 0.5, 2, and 3 h sorbitol treatment. (D) Representative immunofluorescence images showing miniTurbo-YAP-EGFP condensates (green) colocalizes with biotinylated proteins (red) of HeLa cells after 10 min of treatment with 0.2 M sorbitol/500 µM biotin. Data are shown as mean ± SEM; Statistical significance was assessed using unpaired *t* test. *p < 0.05.

Recently, it has been established that liquid-liquid phase separation, a biophysical process of demixing of macromolecules into distinct, co-existing phases (Banani *et al.*, 2017; Shin and Brangwynne, 2017), is a major organizer of the Hippo pathway (Kiang *et al.*, 2024). We are the first to show that when activated by hyperosmotic stress, YAP forms liquid-like droplets/condensates that initially cluster the accessible chromatins, and later activate downstream gene expression (Figure 1A; Cai *et al.*, 2019). The other groups and we also discovered that TAZ (WWTR1, a paralog of YAP), TEAD, and the upstream Hippo kinases form condensates with diverse and distinct functions (Franklin and Guan, 2020; Lu *et al.*, 2020; Wang *et al.*, 2022a; Bonello *et al.*, 2023; Liang *et al.*, 2025). Therefore, understanding the respective components of different Hippo pathway condensates can reveal important clues about how these condensates function under different conditions. YAP condensates can concentrate transcription-related proteins such as TEAD1, TAZ, RNA Pol II, Mediator 1, and BRD4 (Cai *et al.*, 2019; Yu *et al.*, 2021; Hao *et al.*, 2024). However, most other components in YAP condensate remain unknown, hindering the understanding of the detailed organization and functions of YAP condensates.

On the other hand, understanding the components of condensates can provide important insights into therapy. While dysregulated TFs drive many cancers, targeted treatments of these cancers have been lacking since different from kinases, TFs lack deep pockets that drugs can specifically bind to. Recently, it has been found that TFs can form functional biomolecular condensates to drive diseases (Boija *et al.*, 2018; Cho *et al.*, 2018; Sabari *et al.*, 2018). Targeting the specific molecular components and/or interactions among the TF condensates, therefore, emerges as a novel way of treating these diseases (Boija *et al.*, 2021; Cai *et al.*, 2021). YAP initiates and drives numerous types of cancer, and YAP condensates have been implicated in breast cancer, osteosarcoma, and lung adenocarcinoma (Cai *et al.*, 2019; Yu *et al.*, 2021; Hao *et al.*, 2024). Therefore, understanding the components of YAP condensates can reveal novel ways to treat these YAP condensate-implicated cancers.

In this study, we systematically probed, identified, and validated novel components of YAP condensates. Specifically, we found that TCF12 and JUNB are not only clients of YAP condensates, but also contribute to YAP condensate formation through their specific domains. Our results shed light on how YAP carries out its functions and provide insights into future efforts targeting YAP condensate components to treat malignancies driven by YAP.

## RESULTS AND DISCUSSION

### A Proximity-Based Proteomics Approach to Detect YAP-Interacting Proteins

YAP condensates are formed by weak interactions (Cai *et al.*, 2019), making them hard to isolate by centrifugation and immunoprecipitation-based methods. To identify the YAP-interacting proteins and especially those within the YAP condensates, we performed proximity-based proteomics, attaching YAP to a promiscuous biotin ligase miniTurbo (a shortened version of TurboID, which minimizes disruptions to YAP; Branon *et al.*, 2018; Cho *et al.*, 2020) and enhanced green fluorescent protein (EGFP, for visualization of YAP subcellular localization) and expressed it in the HeLa cells. Proteins that are within a few nanometers of YAP will be labeled with exogenous biotin by miniTurbo, and subsequently be pulled down by streptavidin-conjugated beads and identified using mass spectrometry (Figure 1B; Branon *et al.*, 2018; Cho *et al.*, 2020). Fusing YAP with miniTurbo did not affect YAP activities under hyperosmotic stress, evidenced by the formation of liquid-like YAP condensates immediately after sorbitol addition and the activation of the YAP target gene 2 and 3 h after sorbitol addition (Figure 1, C and D), consistent with previous reports (Hong *et al.*, 2017; Cai *et al.*, 2019; Hao *et al.*, 2024). We used immunofluorescence (IF) imaging to verify that miniTurbo successfully biotinylated YAP condensate components, showing high colocalization of YAP with biotin labeled with streptavidin-fluorophore (Figure 1D, Pearson's correlation coefficient = 0.667 ± 0.038). We added biotin to HeLa YAP-miniTurbo-EGFP cells treated with control medium, 10 min sorbitol, and 2 h sorbitol, respectively, digested proteins into peptides, used streptavidin beads to enrich the biotinylated peptides and performed mass spectrometry to identify YAP interactors by matching the biotinylated peptides to respective proteins. HeLa cells without the expression of YAP-miniTurbo-EGFP (WT), similarly treated with 10 min sorbitol and biotin, were used as negative controls.

### Specific components of YAP condensates are detected by mass spectrometry

**FIGURE 2:**
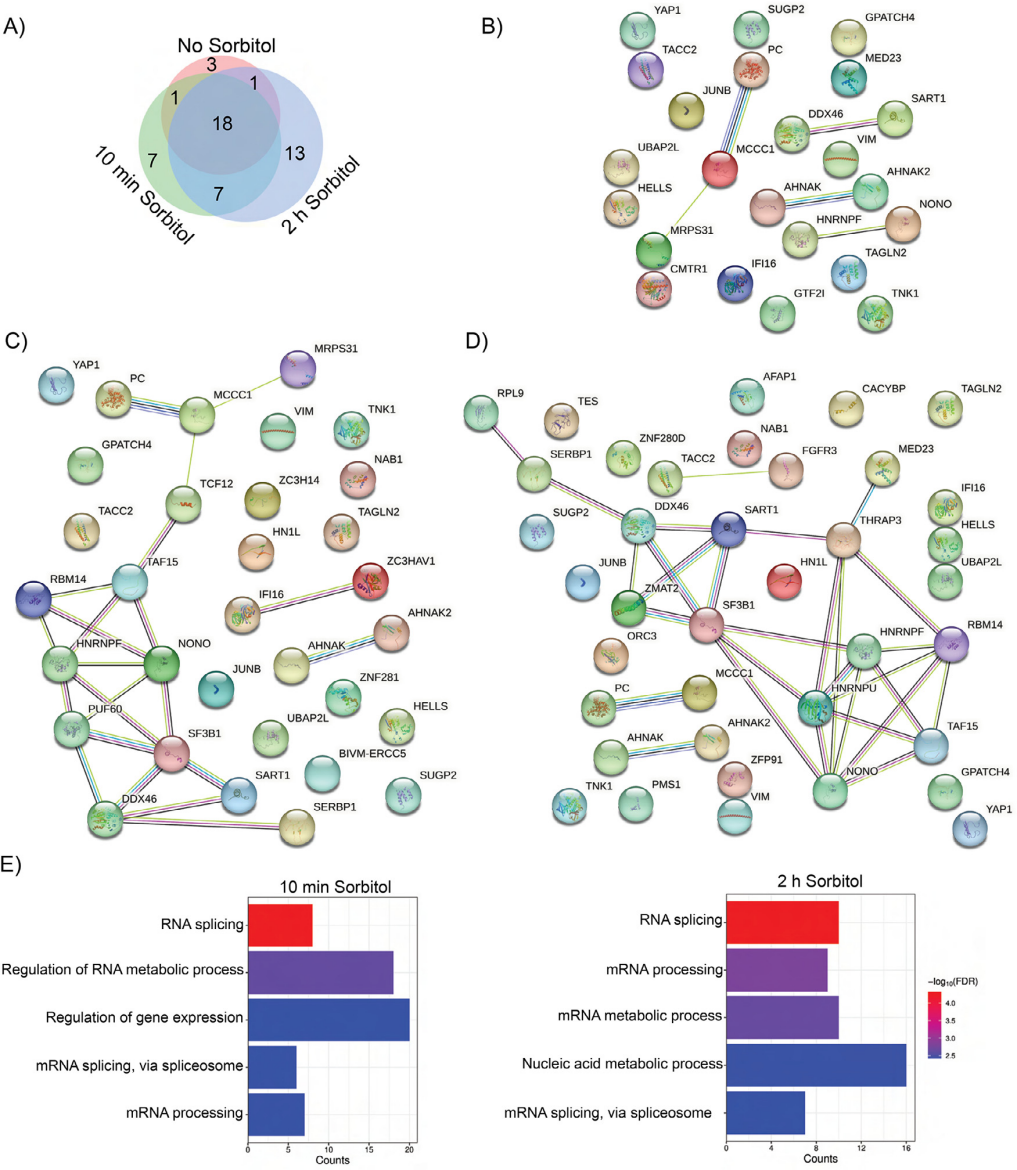
STRING protein-protein interactions (PPI) network maps of biotinylated proteins. (A) Venn diagram demonstrating the number of proteins biotinylated by miniTurbo-YAP-EGFP in HeLa cells treated with no sorbitol, 0.2 M sorbitol for 10 min and 2 h respectively. Each sector denotes the number of proteins uniquely or commonly identified across conditions. (B) STRING PPI analysis of HeLa cells transfected with miniTurbo-YAP-EGFP with 10 min of 500 µM biotin treatment (no sorbitol ctrl). PPI enrichment *p*-value: 0.133 (C) STRING PPI analysis of HeLa cells transfected with miniTurbo-YAP-EGFP with 10 min of 0.2 M sorbitol/500 µM biotin treatment (10 min sample). PPI enrichment *p*-value: 3.56e-07 (D) STRING PPI analysis of HeLa cells transfected with miniTurbo-YAP-EGFP with 2 h of 0.2 M sorbitol/500 µM biotin treatment (2 h sample). PPI enrichment *p*-value: 1.3e-07. (E) Gene Ontology (GO) enrichment analysis of biological process (BP) terms for proteins biotinylated by miniTurbo-YAP-EGFP in HeLa cells treated with 0.2 M sorbitol for 10 min and 2 h respectively. The bar plot shows the top five enriched GO biological processes ranked by statistical significance (−log₁₀ FDR).

Using mass spectrometry, we identified 23 proteins in the no sorbitol control, 33 proteins in 10 min sorbitol, and 39 proteins in 2 h sorbitol-treated samples, respectively (Supplemental Table S1, control, 10 min sorbitol, 2 h sorbitol). On the other hand, only a few peptides were identified in the WT negative control, showing the specificity of our proximity-based proteomics approaches (Supplemental Table S1, WT). As expected, YAP was identified in all three YAP-miniTurbo-EGFP expressing samples, confirming the efficacy of our approach. While some proteins were shared, unique components were identified in each group (Figure 2A), indicating that interactors of YAP evolve with condensation and functional changes. Using STRING database to analyze the protein–protein interaction (PPI) network (Snel *et al.*, 2000), we found that 10 min and 2 h sorbitol samples show distinct, nonrandom clustering, with significant enrichment *p*-values compared with random interactions (Figure 2, B–D, *p*-values = 0.133, 3.56e-07, and 1.3e-07 respectively for control, 10 min and 2 h sorbitol treatments respectively). Interestingly, many of the proteins in the network play roles in RNA processing and are predicted to be nuclear and RNA-binding proteins (Figure 2E; Supplemental Figure S1), consistent with the roles of YAP in transcription. After excluding known carboxylases and giant scaffold proteins, which are abundant cellular proteins likely contaminants, we curated a list of proteins with their known functions (Supplemental Figure S2). Interestingly, more proteins were enriched after 2 h of sorbitol treatment during which YAP target genes have been activated (Cai *et al.*, 2019). Consistently, the Gene Ontology (GO) term “nucleic acid metabolic process” was highly enriched after 2 hrs of sorbitol treatment, and included unique proteins such as THRAP3 (thyroid hormone receptor-associated protein 3, a transcriptional coactivator) and ZMAT2 (zinc finger matrin-type 2, a component of the spliceosome; Figure 2E; Supplemental Figure S2), indicating that YAP condensates may engage with transcriptional cofactors and the splicing machinery to accelerate downstream gene expression. Among all the proteins interacting with YAP after 2 h of sorbitol treatment, ZNF280D (zinc finger protein 280D) and ZFP91 (zinc finger protein 91) are TFs that can activate MAP3K14 (mitogen-activated protein kinase 14) and NIK (NF-κB-inducing kinase), respectively (Jin *et al.*, 2010). While outside the scope of the current study, future studies will test how these factors may drive gene expression for surviving hyperosmotic stress.

### TCF12 and JUNB are Novel Components of YAP Condensates

**FIGURE 3:**
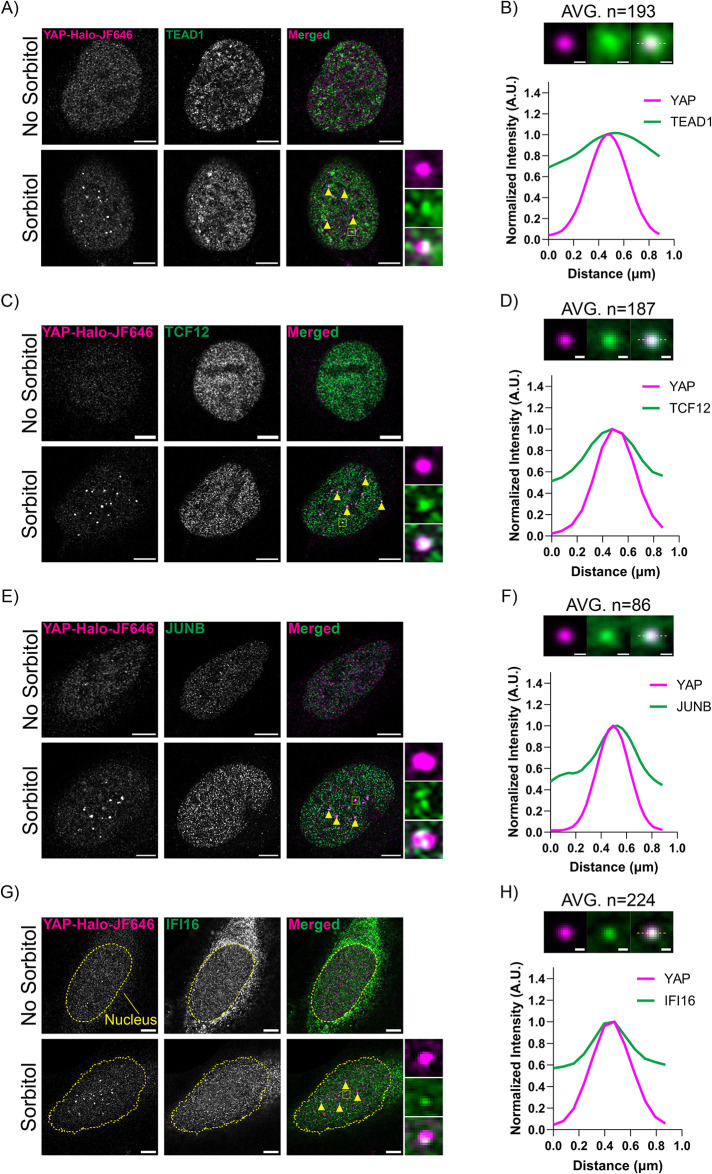
Identification of endogenous proteins enriched in endogenous YAP condensates. (A) Representative immunofluorescence images of U-2 OS YAP-HaloTag cells, with JF646 Halo Dye and TEAD1 antibody staining, after control (no sorbitol) and 5 min 0.2 M sorbitol treatments. Arrows denote a few YAP condensates. Insets are magnified views of the boxed region, showing accumulation of TEAD1 on a YAP condensate. (B) Average Intensity (AVG) images centering on individual YAP condensates (magenta) formed after 5 min 0.2M sorbitol treatment, showing enrichment of TEAD1 signal (green) in the center. Line plots (below) show normalized intensity of indicated channels along the yellow dotted line drawn through the merged averaged image (above). (C–H) Similar to (A and B) but show enrichment of TCF12 (C and D), JUNB (E and F), and IFI16 (G and H) in YAP condensates formed after 5 min 0.2M sorbitol treatments. In (A, C, E, G), scale bars = 5 µm, scale bars = 0.3 µm for inset; (B, D, F, H), scale bars = 0.3 µm. Yellow dashed lines denote nuclear boundaries.

We then focused on the YAP-interacting proteins at 10 min of sorbitol treatment to find YAP condensate components. Among the 33 candidate proteins enriched in the 10 min sorbitol treated sample, 8 candidates have transcription and RNA splicing-related activities (Supplemental Figure S2). To verify that these proteins indeed concentrate in endogenous YAP condensates, we performed IF on these candidates in a well-characterized U-2 OS YAP-HaloTag cell line, in which a HaloTag is knocked into the YAP locus to track endogenous YAP dynamics (Cai *et al.*, 2019; Hao *et al.*, 2024). As a positive control, upon hyperosmotic shock, endogenous YAP formed nuclear condensates that concentrated TEAD1 as reported before (Figure 3, A and B). Among the candidates, while TCF12 (transcription factor 12) and JUNB (transcription factor JunB) remained nuclear both before and after sorbitol treatment, IFI16 (interferon alpha-inducible protein 6) underwent a striking relocalization after sorbitol treatment, shifting from predominantly cytoplasmic and peri-nuclear to nuclear-localized (Figure 3, C, E, and G). After sorbitol treatment and YAP condensate formation, TCF12, JUNB, and IFI16 all strongly enriched inside YAP condensates in both individual cells and in averaged images centering on individual YAP condensates (Figure 3, C–H, Table 3), while other candidates were not enriched or depleted from YAP condensates (Supplemental Figure S3, Table 3). TCF12 is a basic helix-loop-helix (bHLH) TF that drives lineage-specific gene expression (Zhang *et al.*, 1991); JUNB is an AP-1 family TF that homodimerizes or heterodimerizes with other AP-1 family TFs to mediate transcription (Ren *et al.*, 2023); and IFI16 is an interferon-stimulated protein that mediates innate immune response and is thus protective against tumors and viruses (Unterholzner *et al.*, 2010; Ka *et al.*, 2021). Among them, only JUNB has been shown to interact with the YAP/TEAD complex (Zanconato *et al.*, 2015; He *et al.*, 2021), and none of them were previously implicated to be endogenous components of a YAP condensate. We went ahead to test if they are mere clients of YAP condensates or otherwise have functions in YAP condensate formation.

### TCF12 and JUNB are Important for YAP Condensate Formation

**FIGURE 4:**
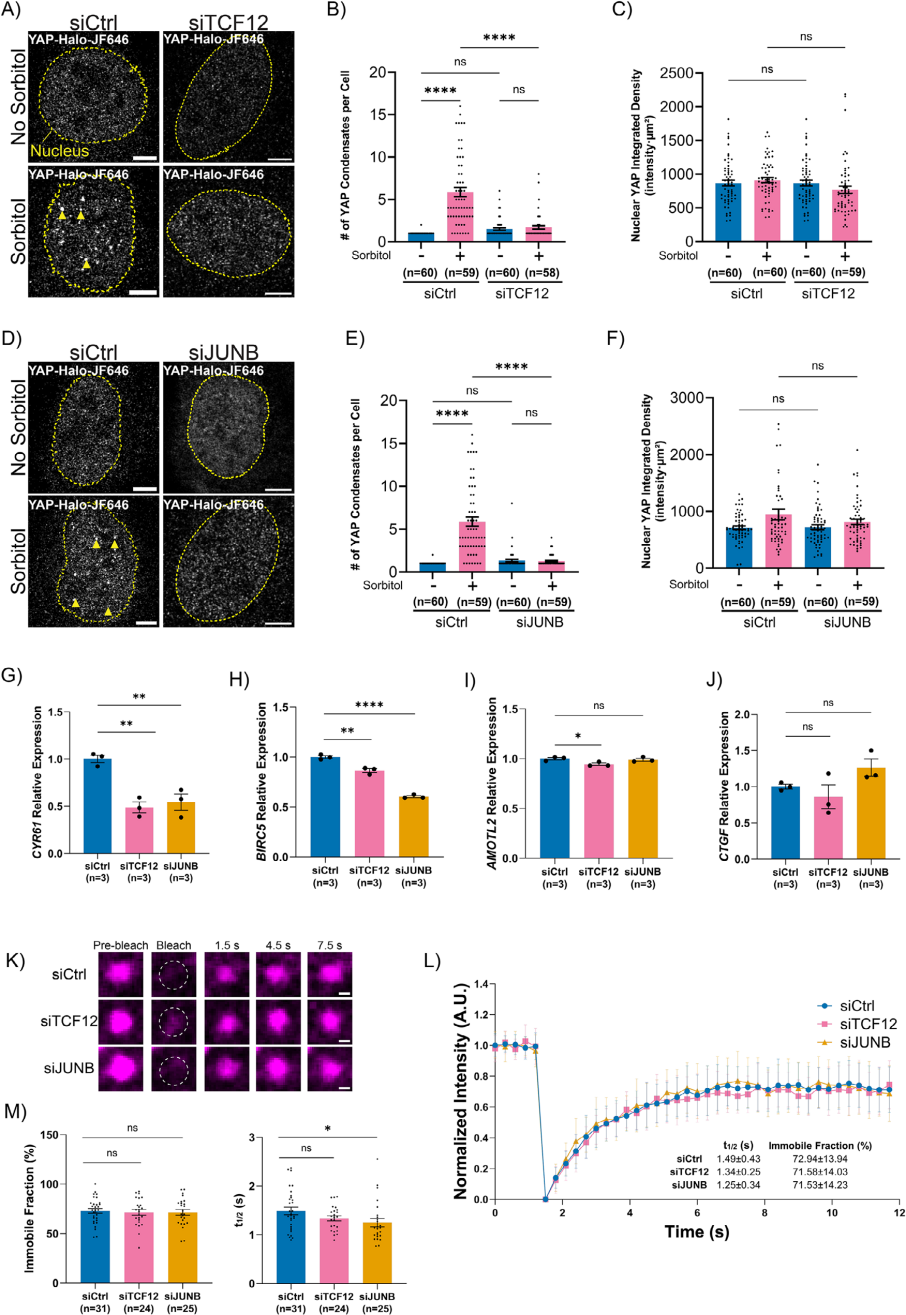
Knockdown of TCF12 and JUNB inhibited YAP condensate formation. U-2 OS YAP-HaloTag cells were treated with control siRNA (siCtrl), siRNA targeting TCF12 (siTCF12) or JUNB (siJUNB) respectively. (A and D) Representative immunofluorescence images showing YAP localization. Yellow dashed lines mark nuclear boundaries. Arrows denote a few YAP condensates. Scale bar = 5 µm. (B, E) Quantification of YAP condensates per cell in siCtrl versus (B) siTCF12 and (E) siJUNB groups under untreated or 5 min 0.2 M sorbitol conditions. (C and F) Nuclear YAP integrated density in siCtrl versus (C) siTCF12 and (F) siJUNB groups under the same conditions. (G–J) Relative expression levels of YAP target genes (G) CYR61, (H) BIRC5, (I) AMOTL2, and (J) CTGF, were quantified by RT-qPCR from siCtrl versus siTCF12 and siJUNB treated cells. (K) Representative imaging showing FRAP recovery of YAP-Halo condensate (labeled with JF646 Halo Dye) in siCtrl, siTCF12, and siJUNB cells, scale bars = 0.3 µm. (L) FRAP recovery curve (*n* = 31 for siCtrl, *n* = 24 for siTCF12, *n* = 25 for JUNB). (M) Quantifications of immobile fraction (left) and t_1/2_ (right) from FRAP. Statistical significance was assessed using ordinary one-way ANOVA (B, C, E, and F), or unpaired *t* test (G, H, I, J, and M); ns, not significant; * *p* < 0.05; ** *p* < 0.01; **** *p* < 0.0001. Data are shown as mean ± SEM.

We first investigated whether TCF12 is important for YAP condensate formation. We used siRNA to specifically knockdown (k.d.) TCF12, achieving a 97% depletion of TCF12 protein (Supplemental Figure S4A). Intriguingly, compared with cells treated with control siRNA, which form prominent YAP condensates after sorbitol treatment, those treated with siTCF12 formed significantly fewer YAP condensates (Figure 4, A and B). Importantly, neither the nuclear accumulation nor the total protein amount of YAP was significantly affected after TCF12 k.d. (Figure 4C; Supplemental Figure S4C). Therefore, TCF12 is important for the regulation of YAP condensation. We then tested the role of JUNB in YAP condensation. We found that, similar to TCF12, a 93% depletion of JUNB through siRNA (Supplemental Figure S4B) inhibited YAP condensation (Figure 4, D and E) without affecting nuclear or total YAP levels, indicating that JUNB is also important for seeding YAP condensate formation (Figure 4F; Supplemental Figure S4C). Strikingly, TCF12 or JUNB knockdown impaired YAP transcriptional activity, reducing expression of the YAP target genes *CYR61* and *BIRC5*, and additionally lowering *AMOTL2* levels upon siTCF12 treatment (Figure 4, G–I, Table 2). The level of *CTGF*, however, is not affected after TCF12 or JUNB k.d., possibly because *CTGF* expression is mainly driven by YAP outside of condensates in the U-2 OS cells (Figure 4J, Table 2). Lastly, JUNB also contributes to the stability of YAP condensates, as knocking it down increased the fluidity of the remaining YAP condensates (evidenced by a decrease of t_1/2_ in fluorescence recovery after photobleaching experiments), while TCF12 k.d. did not have a similar effect (Figure 4, K–M). On the other hand, we cannot test the necessity of IFI16 in YAP condensate formation: while 2 d of k.d. of IFI16 did not affect IFI16 levels, 3 d of k.d. affected cell viability (data not shown). In summary, we have found that the newly identified YAP condensate components TCF12 and JUNB are important for YAP condensate formation and their transcriptional activities.

### TCF12 and JUNB use Specific Domains to Interact with YAP and Incorporate into YAP Condensates

**FIGURE 5:**
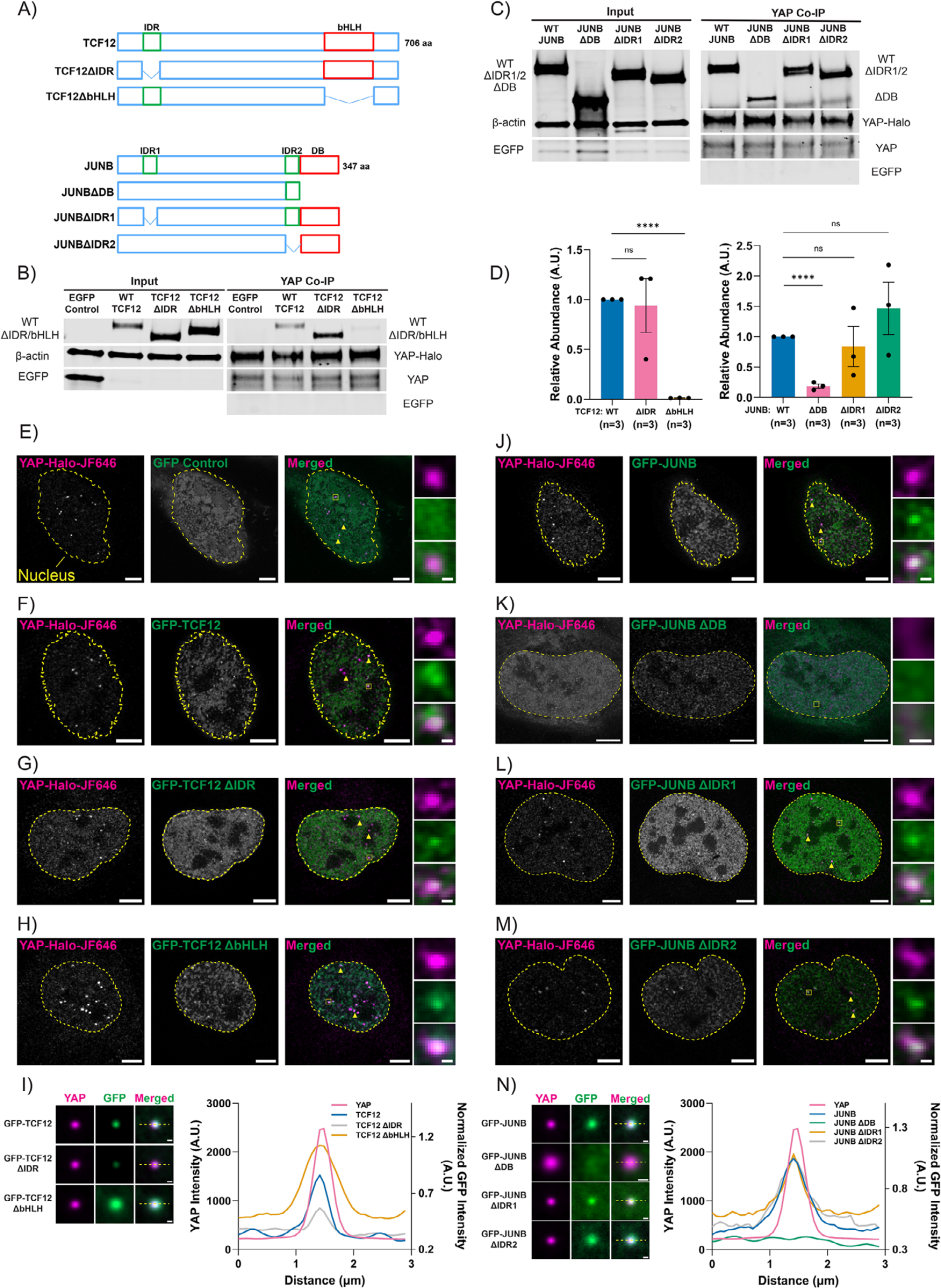
Investigating the functional domains in TCF12 and JUNB for YAP binding and YAP condensate incorporation. (A) Schematic illustration of TCF12 and JUNB and their respective domain deletions. (B and C) Co-immunoprecipitation assays demonstrate YAP interactions with (B) TCF12 and TCF12 mutants as well as (C) JUNB and JUNB mutants. (D) Quantifications of protein abundance for mutants of TCF12 (left) and JUNB (right) in the YAP Co-IP fraction relative to WT TCF12 or JUNB. (E–M) Representative immunofluorescence images of U-2 OS YAP-HaloTag cells stained with JF646 Halo Dye, and transfected with (E) GFP vector (GFP control), (F–H) GFP tagged WT TCF12 and TCF12 mutants, and (J–M) GFP-tagged WT JUNB and JUNB mutants, after 5 min 0.2 M sorbitol treatments. Arrows denote a few YAP condensates. Insets are magnified views of the boxed region. Yellow dashed lines denote nuclear boundaries. (I, left) Averaged images centering on individual YAP condensate (magenta) formed after 5 min 0.2M sorbitol treatment and GFP-tagged WT TCF12, as well as mutants relative to YAP condensate. (I, right) Line plots show raw YAP intensity and GFP-TCF12 and mutants intensity normalized to GFP control along the yellow dotted line drawn through the merged averaged image (*n* = 217 for TCF12, *n* = 267 for TCF12ΔIDR, *n* = 433 for TCF12ΔbHLH). (N) Similar to I, but show GFP-tagged WT-JUNB, and mutants (*n* = 234 for JUNB, *n* = 20 for JUNBΔDB, *n* = 161 for JUNBΔIDR1, *n* = 154 for JUNBΔIDR2). In (E–H, J–M), scale bars = 5 µm, scale bars = 0.3 µm for inset; (I, N), scale bars = 0.3 µm. Yellow dashed lines denote nuclear boundaries. Statistical significance was assessed using unpaired t-test (D). ns, not significant; **** *p* < 0.0001. Data are shown as mean ± SEM.

To elucidate the molecular mechanisms by which TCF12 and JUNB regulate YAP condensate formation, we assessed a series of GFP-tagged domain deletion mutants for their interactions with endogenous YAP and their recruitment into condensates (Figure 5A). As expected, a GFP-only negative control failed to bind YAP or enrich within YAP condensates (Figure 5, B, C, and E). TCF12 consists of an N-terminal intrinsically disordered region (IDR) and a C-terminal DNA-binding bHLH domain (Figure 5A). While wild-type GFP-TCF12 directly bound endogenous YAP and enriched at YAP condensates (Figure 5, B, F, and I), deleting the IDR (ΔIDR, Table 1) produced a decoupling effect: direct TCF12–YAP binding did not significantly change, yet condensate enrichment was significantly reduced (Figure 5, B, D, G, and I). This indicates that while the IDR may be dispensable for direct YAP binding, it is essential for incorporation into the condensate phase. Conversely, removing the bHLH domain (ΔbHLH, Table 1) disrupted TCF12–YAP interactions and yet enhanced TCF12 recruitment and promoted the assembly of larger, more numerous YAP condensates (Figure 5, B, D, H, and I; Supplemental Figure S4, D and E). These results indicate that, interestingly, TCF12 utilizes different domains for direct YAP binding (bHLH) and YAP condensate incorporation (IDR).

We performed a parallel analysis for JUNB, which contains two IDRs and a C-terminal DNA-binding (DB) domain (Figure 5A). In contrast to TCF12, JUNB's regulation of YAP appears primarily dependent on its DNA-binding capacity. Deletion of the DB domain (ΔDB, Table 1) drastically reduced JUNB–YAP interactions and inhibited condensate formation (Figure 5, C, D, K, and N; Supplemental Figure S4, F and G). This suggests that YAP condensates nucleate near JUNB-bound genomic sites, and that the JUNBΔDB mutant acts as a dominant negative that prevents localized YAP concentration and condensation. On the other hand, neither IDR1 nor IDR2 was required for direct YAP binding, condensate incorporation, or the regulation of condensate morphology (Figure 5, C, D, L, M, N; Supplemental Figure S4, F and G, Table 1). Taken together, these results indicate that the JUNB uses the DB for direct YAP binding and incorporation into YAP condensates.

Biomolecular condensates are heterogeneous, multicomponent systems that can have evolving functions over time. Understanding their components will reveal important information about how condensates organize spatiotemporally to achieve specific functions. We have previously found that YAP condensates formed under hyperosmotic stress initially cluster accessible chromatin regions and later activate transcription (Cai *et al.*, 2019). In this study, using a proximity-based proteomics approach, we have identified novel components of YAP condensates. The AP-1 complex is known to colocalize with YAP/TEAD on genomic sites to drive proliferative gene expression (Zanconato *et al.*, 2015; Park *et al.*, 2020). AP-1 consists of heterodimers of JUN (c-JUN, JUNB) and FOS. Among them, JUNB has been shown to directly interact with YAP and TAZ through their WW domains (He *et al.*, 2021). In our study, we found that JUNB is not only a component of YAP condensate but is also critical for YAP condensate formation. This finding adds another important insight into the convergence of AP-1 and YAP/TEAD signaling. Under hyperosmotic stress, co-segregation of YAP and JUNB to the same condensate may mediate the expression of survival-related genes co-activated by the two pathways. TCF12 is another newly identified factor involved in YAP condensate formation. The canonical function of TCF12 is to drive muscle and neuronal differentiation during development by heterodimerizing with other bHLH TFs (Mesman and Smidt, 2017; Wang *et al.*, 2022b). Despite no existing evidence of direct interactions with Hippo pathway components, TCF12 has been implicated in driving the progression of various cancers, such as melanoma and ovarian cancers (Gao *et al.*, 2019; Tian *et al.*, 2023), in which YAP also plays essential roles. It will be interesting to investigate if TCF12 and YAP interact within the same condensates in these cancers. Furthermore, since TCF12 drives YAP condensate formation, it is worth testing if disrupting TCF12 indeed can inhibit YAP condensate formation in these cancers.

### Limitations of the Study

Our IFI16 k.d. experiments killed the cells, so we cannot access the roles of IFI16 in YAP condensation. The interaction studies of YAP with TCF12, JUNB, and their respective mutants were done in the presence of endogenous TCF12 and JUNB.

## MATERIALS AND METHODS

Protocols and information about reagents are available from the corresponding author, D. C. (danfeng.cai@jhu.edu), to others upon request. Novel materials and reagents in this manuscript will be available for distribution to other researchers once published.

### Cell Cultures

All cell lines used in this study were cultured at 37°C under 5% CO_2_ in Dulbecco's Modified Eagle's Medium (DMEM; 15-013-CV, Corning) supplemented with 10% fetal bovine serum (FBS; Life Technologies, 26140079), 10,000 U/ml (1%) penicillin/streptomycin (Life Technologies, 15140122) and 2 mM (1%) GlutaMAX-l (Life Technologies, 35050061).

### Cell Lines

***HeLa cells***. HeLa cells were gifted by Dr. Anthony K. L. Leung at Johns Hopkins Bloomberg School of Public Health.

***U-2 OS YAP-HaloTag cell line***. U-2 OS YAP-HaloTag cell line was generated previously by Dr. Danfeng Cai as described (Cai *et al.*, 2019).

Cell lines are tested for mycoplasma when the cell line is initially obtained, and then routinely (i.e., every three to six months) thereafter using the Mycoalert detection kit (LT07-318, Lonza) and at the JHU Tissue Culture Facility. Cell line authenticity is established through the JHU Vironomics Core Facility upon receipt of cell lines and before freezing down stocks. Authentication is performed using the Ion Torrent Precision ID Global Filter NGS STR Panel of forensically relevant short tandem repeats (STRs) from genomic DNA.

### Plasmid Constructions

For cloning WT TCF12 into pcDNA3.1-GFP-Flag-EPEA vector, pcDNA3.1-GFP-Flag-JUNB-EPEA (Addgene, 160743) was linearized by PCR (primers are listed below) using Q5 High-Fidelity 2X Master Mix (New England Biolabs, M0492S) on C1000 Touch Thermal Cycler (Bio-Rad, 1851148) according to manufacturer's instructions. The PCR product was verified using gel-electrophoresis with 0.8% gel made from UltraPure Agarose (Invitrogen, 16500500) and 1X TAE buffer diluted from 50X TAE buffer (Quality Biological, 351-008-131).TCF12 DNA fragment (synthesized by Twist Bioscience) was cloned into linearized pcDNA3.1-GFP-Flag-EPEA vector using NEBuilder® HiFi DNA Assembly Cloning Kit (New England Biolabs, E5520S) according to manufacturer instructions.

For generation of plasmid carrying truncated JUNB, TCF12, and EGFP control, pcDNA3.1-GFP-Flag-JunB-EPEA (Addgene, 160743) and pcDNA3.1-GFP-Flag-TCF12-EPEA were linearized and verified in the same manner as mentioned above (corresponding primers using for each mutagenesis are listed below) Verified PCR products were ligated using Q5 Site-Directed Mutagenesis Kit (New England Biolabs, E0554) according to manufacturer instructions.

**TABLE 1:** Primers for Q5 site-directed mutagenesis.

Plasmid	Forward (5′ → 3′)	Reverse (5′ → 3′)
pcDNA3.1-GFP-Flag-JunBΔIDR1-EPEA	GTTGACCGCCAGGCTCGGTTT	AAGCTCGCCTCTTCGGAGCT
pcDNA3.1-GFP-Flag-JunBΔIDR2-EPEA	ATCAACATGGAAGACCAAGAGCGCATCA	CGCGTGTGGGAGGTAGCTG
pcDNA3.1-GFP-Flag-JunBΔDB-EPEA	GCATAACTCGAGTCTAGAGGGCCC	CGCGTGTGGGAGGTAGCTG
pcDNA3.1-GFP-Flag-EPEA	GCATAACTCGAGTCTAGAGGGCCC	CTTATCGTCGTCATCCTTGTAGTCTGGCG
pcDNA3.1-GFP-Flag-TCF12ΔIDR-EPEA	GATACTGGATTACCAGGCTGT	ACTGAAGTCCAGTAGGTCG
pcDNA3.1-GFP-Flag-JunBΔbHLH-EPEA	GAACAGCAAGTCAGAGAGA	CTTCTCCCTTTCTATCTTCTGT
pcDNA3.1-GFP-Flag-TCF12-EPEA	AACAACGCATGGCCGCTA	GGGATCAAGCGCTGCAGA
pcDNA3.1-GFP-Flag-EPEA (For TCF12 cloning only)	CTCGAGTCTAGAGGGCCCGTTTAAAC	CCGTTCCTTGGCGGCG

### Transfection

DNA plasmid transfection was performed by Lipofectamine 3000 Transfection Reagent (Thermo Fisher, L3000015) according to the manufacturer's instructions for 16-24 h. The following DNA plasmid was used in this study:

1. miniTurbo-YAP_pcDNA3.1(+)-C-eGFP (synthesized by Genscript), 2. pcDNA3.1-GFP-Flag-JunB-EPEA (Addgene, 160743), 3. pcDNA3.1-GFP-Flag-EPEA, 4. pcDNA3.1-GFP-Flag-TCF12-EPEA, 5. pcDNA3.1-GFP-Flag-JunBΔIDR1-EPEA, 6. pcDNA3.1-GFP-Flag-JunBΔIDR2-EPEA, 7. pcDNA3.1-GFP-Flag-JunBΔDB-EPEA, 8. pcDNA3.1-GFP-Flag-TCF12ΔIDR-EPEA, 9. pcDNA3.1-GFP-Flag-TCF12ΔbHLH-EPEA. Plasmids 3-9 were all modified from Addgene, 160743.

siRNA transfection was conducted using the Lipofectamine RNAiMAX transfection reagent (Thermo Fisher,13778075) according to manufacturer instructions for 24–72 h.

The following siRNAs were used in this study:

1. Scrambled negative control siRNA (Thermo Fisher, AM4611),
2. JunB siRNA (Thermo Fisher, Silencer Select s7661),
3. ON-TARGETplus Human TCF12 siRNA (Dharmacon, Horizon Discovery, L-006356-00-0005),
4. ON-TARGETplus Human IFI16 siRNA (Dharmacon, Horizon Discovery, L-020004-00-0005)

### Protein Extraction

For samples directly used for western blot (WB), whole cell proteins were extracted by lysing cells in cold RIPA lysis buffer (Thermo Fisher, 89900) with cOmplete Mini Protease Inhibitor Cocktail (Sigma Aldrich, 11836170001). After 1 h incubated on ice, insoluble debris was removed by centrifugation at 21,000 × g for 15 min at 4°C. For samples used for co-immunoprecipitation (Co-IP), whole cell proteins were extracted by lysing cells in cold Pierce IP Lysis Buffer (Thermo Scientific, 87787) with cOmplete Mini Protease Inhibitor Cocktail (Sigma Aldrich, 11836170001). After 15 mins incubation on ice, Insoluble debris was removed by centrifugation at 21,000 × g for 15 min at 4°C. Nuclear Proteins were extracted via the EpiXtract Nuclear Protein Isolation kit (Enzo Life Sciences, ENZ-45016-0100) following the manufacturer's instructions. The concentration of proteins was measured with Pierce 660 (Thermo Scientific, 22660) and Biotek Synergy HT Multi-Mode Microplate Reader.

### TurboID and Mass Spectrometry

HeLa cells were either transfected with miniTurbo-YAP-EGFP for 24 h or left untransfected to serve as a negative control. Before nuclear protein extraction, the cells were subjected to one of four proximity labeling conditions: (1) untransfected cells treated with 0.2 M sorbitol and 500 µM biotin for 10 min (negative control), (2) transfected cells treated with 500 µM biotin for 10 min (no sorbitol control), (3) transfected cells treated with 0.2 M sorbitol and 500 µM biotin for 10 min, or (4) transfected cells treated with 0.2 M sorbitol for 2 h, with the addition of 500 µM biotin during the final 10 min of incubation. ∼200 µg of nuclear proteins were digested into peptides with trypsin. The peptides were then dissolved in RIPA lysis buffer (Thermo Fisher, 89900) with cOmplete Mini Protease Inhibitor Cocktail (11836170001, Sigma Aldrich; Sigma Aldrich, 11836170001) and incubated with Pierce Streptavidin Magnetic Beads (Thermo Fisher, 88817) overnight at 4°C using a rotator. The next day, the beads were washed twice with RIPA lysis buffer, once with 1.0 M Potassium chloride, once with 50 mM TEAB buffer (Thermo Scientific, 90114), twice with RIPA lysis buffer, three times with PBS, and dried under vacuum. The dry beads were sent to the Mass Spectrometry and Proteomics Core at Johns Hopkins School of Medicine, eluted with 1,1,1,3,3,3-hexafluoro-2-propanol (HFIP), and subjected to standard Mass Spectrometry.

### Protein–Protein Interaction Analysis

The biotinylated peptides identified from each sample were matched to proteins using Proteome Discoverer Software (Thermo Fisher) and separately uploaded to the STRING database for protein-protein interaction (PPI) and Gene Ontology analysis.

### RT-qPCR

Total RNA was isolated from U-2 OS YAP-HaloTag cells using the Direct-zol RNA MiniPrep kit (Zymo Research, R2052) and converted to complementary DNA using the High-Capacity RNA-to-cDNA reverse transcription kit (Thermo Fisher, 4387406). The RT-qPCR was performed on a QuantStudio 3 Real-Time PCR Instrument using PowerUp SYBR Green Master Mix (Thermo Fisher, A25742). Three biological replicates for each target gene under each treatment were performed.

**TABLE 2:** Primers for RT-qPCR.

Gene	Forward (5′ → 3′)	Reverse (5′ → 3′)
***CYR61***	CCTCGGCTGGTCAAAGTTAC	TTTCTCGTCAACTCCACCTC
***BIRC5***	CCACTGAGAACGAGCCAGACTT	GTATTACAGGCGTAAGCCACCG
***AMOTL2***	CACAGGCATCAGGAGATGGAAAG	GCGCTGCTGAAGGACCTTG
***CTGF***	AGGAGTGGGTGTGTGACGA	CCAGGCAGTTGGCTCTAATC

### Immunoblot

Proteins (10–30 ng) were mixed with 4x Protein loading buffer (LI-COR Biosciences, 928-40004; with addition of 10% 2-Mercaptoethanol (Millipore Sigma, M3148)) and the samples were boiled at 95°C for 10 min. For western blotting, proteins were separated at 10% Mini-PROTEAN (Bio-Rad, 4561033) gels or 4-12% gradient gel (GenScript, M00654). Proteins were then transferred to a nitrocellulose membrane (0.22 µm, LI-COR Biosciences, 926-31090) at 100 V for 60 min using ice-cold transfer buffer (25 mM Tris, 192 mM glycine, pH 8.3, 20% methanol vol/vol%) or to a Trans-Blot Turbo RTA Mini 0.2 µm Nitrocellulose membrane (Bio-Rad, 1704270) using Bio-Rad Trans-Blot Turbo Transfer System (30 min standard protocol). All membranes were blocked with 5% BSA or non-fat milk in 1X Tris-buffered saline (TBS) at room temperature for 1 h and then incubated with primary antibodies with 3% BSA or non-fat milk in 1X Tris-buffered saline and 1% Tween-20 (TBS-T) at 4°C overnight. After washing three times with TBST, membranes were incubated with fluorescent-conjugated secondary antibodies diluted with 3% BSA or non-fat milk in TBST at room temperature for 2 h. Then, signals were detected, and intensities were measured and analyzed using Odyssey Imaging Systems and Image Studio (LI-COR Biosciences). Three biological replicates for each target protein under each treatment were performed. For statistical quantification, all target protein bands were normalized to β-actin by taking the band intensity of the target protein to β-actin ratio, and then the relative abundance was calculated.

### Immunofluorescence

Cells were plated on #1.5 round glass coverslips coverslips pre-coated with fibronectin (7.5 µg/ml; MilliporeSigma, FC010) and grown for 16–24 h, fixed with 4% formaldehyde (12606S, Cell Signaling Technology) in PBS for 10 min, permeabilized with 0.1% Triton X-100 in PBS for 15 min, and blocked with 3% BSA in PBS at room temperature for 1 h. The coverslips were then incubated overnight with primary antibodies diluted with 1% BSA at 4°C. After washing three times with PBS, the coverslips were incubated with Alexa Fluor-conjugated secondary antibodies diluted with 1% BSA for 1 h at RT. DNA was stained with Hoechst 33342 (Thermo Fisher, 62249, 1:1000 dilution in PBS).

### Co-Immunoprecipitation

For each sample, 50 ul of Dynabeads Protein A/G for Immunoprecipitation (Invitrogen, 10001D (Protein A), 10003D (Protein G), A for anti rb antibodies, G for anti ms antibodies) were placed on magnetic rack (Sergi Lab Supplies, 1005b) pre-washed with cold PBS, and cold Pierce IP Lysis Buffer, and then resuspended with 50 ul Pierce IP Lysis Buffer. Resuspended Dynabead was then incubated with primary antibodies at 4°C for 2 h with gentle shaking. After incubation, Dynabeads were washed with Pierce IP Lysis Buffer three times and then resuspended with protein lysis and incubated at 4°C overnight with gentle shaking.

Following incubation, dynabeads and protein lysis mixture were placed on a magnetic rack, and the supernatant was collected as the post-IP sample. The dynabeads were washed three times with Pierce IP Lysis Buffer. During each wash, the beads were resuspended in lysis buffer and gently shaken at 4°C for 5 min. After washing, Dynabeads were resuspended with 50 ul 4x Protein loading buffer (with addition of 10% 2-Mercaptoethanol) and boiled at 95°C for 10 min. Following that, boiled samples were placed on a magnetic rack, and the supernatant was collected as Co-IP sample, which was ready for loading on SDS–PAGE. Co-IP samples were then analyzed with immunoblotting. During immunoblotting, the Co-IP samples were sequentially imaged by incubating the membrane with α-YAP, α-β-actin, and then secondary antibody first. After first blotting and imaging, the membrane was incubated with α-GFP and then with the secondary antibody. For statistical quantification, the relative abundance of the target protein in the Co-IP fraction was calculated with the equation below.




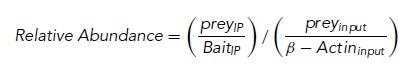




**TABLE 3:** List of antibodies for Western blot (WB), Immunofluorescence (IF), and Co-IP.

Antibody	Vendor	Identifier	Dilution
YAP (D8H1X) XP Rabbit mAb	Cell Signaling	14074	1:1000 (WB), 1:150 (IF), 1: 50 (Co-IP)
YAP (1A12) Mouse mAb	Cell Signaling	12395	1:1000 (WB)
Anti-*β*-Actin (ACTB) Antibody	MilliporeSigma	A1978–100UL	1:10000 (WB)
TCF12/HEB (E8R5E) Rabbit mAb	Cell Signaling	95359	1:10000 (WB), 1:500 (IF)
JunB (C37F9) Rabbit mAb	Cell Signaling	3753	1:10000 (WB), 1:400 (IF)
IFI16 Monoclonal Antibody (2I3D7)	Thermo Fisher	MA5–49174	1:10000 (WB), 1:100 (IF)
NONO Monoclonal Antibody	Thermo Fisher	PA5–27408	1:500 (IF)
TAF15 Monoclonal Antibody (8TA–2B10)	Thermo Fisher	MA3–078	1:250 (IF)
TEAD1 (D9 × 2L) Rabbit mAb	Cell Signaling	12292	1:200 (IF)
DDX46 Polyclonal Antibody	Proteintech	16927–1–AP	1:100 (IF)
SF3B1 Recombinant Rabbit Monoclonal Antibody (6C7)	Thermo Fisher	MA5–49915	1:200 (IF)
SART1 Polyclonal Antibody	Proteintech	22675–1–AP	1:200 (IF)
IRDye 680RD Streptavidin	LI–COR Biosciences	926–68079	1:3000 (WB)
IRDye 680RD Goat anti–Mouse IgG Secondary Antibody	LI–COR Biosciences	926–68070	1:10000 (WB)
IRDye 800CW Goat anti–Rabbit IgG Secondary Antibody	LI–COR Biosciences	926–32211	1:10000 (WB)
Goat anti–Mouse IgG (H+L) Highly Cross–Adsorbed Secondary Antibody, Alexa Fluor 568	Thermo Fisher	A–11031	1:1000 (IF)
Goat anti–Mouse IgG (H+L) Cross–Adsorbed Secondary Antibody, Alexa Fluor 647	Thermo Fisher	A–21235	1:1000 (IF)
Goat anti–Rabbit IgG (H+L) Cross–Adsorbed Secondary Antibody, Alexa Fluor 568	Thermo Fisher	A–11011	1:1000 (IF)
Goat anti–Rabbit IgG (H+L) Highly Cross–Adsorbed Secondary Antibody, Alexa Fluor 647	Thermo Fisher	A–21245	1:1000 (IF)

### HaloTag Staining

Immediately before fixation, U-2 OS YAP-HaloTag cells were incubated with Janelia Fluor 646 (JF646) HaloTag Ligand (Promega, GA1120, 1:1000 dilution) diluted in cell culture media at room temperature for 30 mins.

### Fixed-Cell Airyscan Imaging

Airyscan imaging was conducted with a Zeiss LSM900 equipped with an Airyscan 2 detector using 63x oil objective, followed by Airyscan processing (2D, default settings) in Zeiss Zen software. For imaging and quantification, at least 20 fields of view per coverslip were randomly chosen by 405 nm-illuminated Hoechst-stained channel or 488 nm-EGFP channels for TCF12 and JUNB truncation experiments. At least three independent biological replicates were quantified for each treatment type.

### Live-Cell Imaging and Fluorescence Recovery after Photobleaching (FRAP)

U-2 OS YAP–HaloTag cells were plated into eight-well LabTek chambered coverglass dishes (Life Technologies, 155409PK). After staining with JF646 dye, the media was replaced with FluoroBrite DMEM Complete Medium (Life Technologies, A1896701) supplemented with 10% fetal bovine serum (FBS; Life Technologies, 26140079), 10,000 U/ml (1%) penicillin/streptomycin (Life Technologies, 15140122), and 2 mM (1%) GlutaMAX-l (Life Technologies, 35050061).

In total, 0.2 M sorbitol treatment was applied 5 min before the FRAP experiment. The region of interest was selected, and FRAP was applied to the entire condensate using the interactive bleaching function in Zen. A total of 50 to 100 rounds of images were taken for each ROI until the fluorescence signal plateaued with 300 ms interval. t1/2 and immobile fraction were analyzed using in-house developed Python scripts on raw images. Immobile fraction was defined as the ratio between the average intensity of the last five images immediately before photobleaching and the average intensity of the last five images in the plateau phase of condensate. t1/2 was defined as the time required for the condensate to restore 50% of the immobile fraction immediately post-photobleaching.

At least 5 ROIs were taken between 5-10 mins after 0.2 M sorbitol treatment, and 5 independent biological replicates were conducted. Raw images were smoothened in Fiji to make a visual representation in Figure 4L.

### Image Analysis

YAP condensate counting and colocalization analysis-To quantify YAP condensate and average YAP signal, along with TEAD1, a potential YAP condensate component, or GFP-TCF/JUNB and their corresponding mutants from a 2D image, a custom ImageJ/Fiji (version 1.54p) macro was used. The nucleus was segmented based on the 405 nm Hoechst-stained channel, and any signal outside the nucleus was cleared out. YAP condensate was identified using the Find Maxima function and counted. A rectangular region of interest (ROI) was generated, centered on YAP condensates, for a colocalization study. Colocalization of two channels is done with the ImageJ BIOP JACoP plugin. Foci averaging was achieved by inputting the foci sequence into Fiji and then taking the Z-project based on average intensity.

Nuclear YAP integrated density quantification-Nuclear YAP integrated density was quantified with a custom ImageJ/Fiji (version 1.54p) macro. The nucleus was segmented based on 405 nm Hoechst-stained channel, and the integrated density for 647 nm JF646 HaloTag ligand-stained channel was measured using the measure function. All the imaging analysis codes used in this paper are uploaded to Github: https://github.com/BMBCaiLab/YAPcomponentpaper_MBoC.git.

### Statistical Analysis

Statistical analysis was performed using GraphPad Prism software.

### Gene Fragments

#### ***MiniTurbo-YAP-EGFP***. 5′ FlagTag linker miniTurbo linker YAP linker EGFP 3′

gattacaaggatgacgacgataagatccgcatcccgctgctgaacgctaaacagattctgggacagctggacggcgggagcgtggcagtcctgcctgtggtcgactccaccaatcagtacctgctggatcgaatcggcgagctgaagagtggggatgcttgcattgcagaatatcagcaggcagggagaggaagcagagggaggaaatggttctctccttttggagctaacctgtacctgagtatgttttggcgcctgaagcggggaccagcagcaatcggcctgggcccggtcatcggaattgtcatggcagaagcgctgcgaaagctgggagcagacaaggtgcgagtcaaatggcccaatgacctgtatctgcaggatagaaagctggcaggcatcctggtggagctggccggaataacaggcgatgctgcacagatcgtcattggcgccgggattaacgtggctatgaggcgcgtggaggaaagcgtggtcaatcagggctggatcacactgcaggaagcagggattaacctggacaggaatactctggccgctatgctgatccgagagctgcgggcagccctggaactgttcgagcaggaaggcctggctccatatctgtcacggtgggagaagctggataacttcatcaatagacccgtgaagctgatcattggggacaaagagattttcgggattagccgggggattgataaacagggagccctgctgctggaacaggacggagttatcaaaccctggatgggcggagaaatcagtctgcggtctgccgaaaagtccggactcagatctcgagctcaagcttcgaattgtacaggatccccgcatatggatcccgggcagcagccgccgcctcaaccggccccccagggccaagggcagccgccttcgcagcccccgcaggggcagggcccgccgtccggacccgggcaaccggcacccgcggcgacccaggcggcgccgcaggcaccccccgccgggcatcagatcgtgcacgtccgcggggactcggagaccgacctggaggcgctcttcaacgccgtcatgaaccccaagacggccaacgtgccccagaccgtgcccatgaggctccggaagctgcccgactccttcttcaagccgccggagcccaaatcccactcccgacaggccagtactgatgcaggcactgcaggagccctgactccacagcatgttcgagctcattcctctccagcttctctgcagttgggagctgtttctcctgggacactgacccccactggagtagtctctggcccagcagctacacccacagctcagcatcttcgacagtcttcttttgagatacctgatgatgtacctctgccagcaggttgggagatggcaaagacatcttctggtcagagatacttcttaaatcacatcgatcagacaacaacatggcaggaccccaggaaggccatgctgtcccagatgaacgtcacagcccccaccagtccaccagtgcagcagaatatgatgaactcggcttcagccatgaaccagagaatcagtcagagtgctccagtgaaacagccaccacccctggctccccagagcccacagggaggcgtcatgggtggcagcaactccaaccagcagcaacagatgcgactgcagcaactgcagatggagaaggagaggctgcggctgaaacagcaagaactgcttcggcaggtgaggccacaggagttagccctgcgtagccagttaccaacactggagcaggatggtgggactcaaaatccagtgtcttctcccgggatgtctcaggaattgagaacaatgacgaccaatagctcagatcctttccttaacagtggcacctatcactctcgagatgagagtacagacagtggactaagcatgagcagctacagtgtccctcgaaccccagatgacttcctgaacagtgtggatgagatggatacaggtgatactatcaaccaaagcaccctgccctcacagcagaaccgtttcccagactaccttgaagccattcctgggacaaatgtggaccttggaacactggaaggagatggaatgaacatagaaggagaggagctgatgccaagtctgcaggaagctttgagttctgacatccttaatgacatggagtctgttttggctgccaccaagctagataaagaaagctttcttacatggttaggtaccgagctcggatccactagtccagtgtggtggaattctgcagatatccagcacagtggcggccgcatgagcaagggcgaggagctgttcaccggggtggtgcccatcctggtcgagctggacggcgacgtaaacggccacaagttcagcgtgtccggcgagggcgagggcgatgccacctacggcaagctgaccctgaagttcatctgcaccaccggcaagctgcccgtgccctggcccaccctcgtgaccaccctgacctacggcgtgcagtgcttcagccgctaccccgaccacatgaagcagcacgacttcttcaagtccgccatgcccgaaggctacgtccaggagcgcaccatcttcttcaaggacgacggcaactacaagacccgcgccgaggtgaagttcgagggcgacaccctggtgaaccgcatcgagctgaagggcatcgacttcaaggaggacggcaacatcctggggcacaagctggagtacaactacaacagccacaacgtctatatcatggccgacaagcagaagaacggcatcaaggtgaacttcaagatccgccacaacatcgaggacggcagcgtgcagctcgccgaccactaccagcagaacacccccatcggcgacggccccgtgctgctgcccgacaaccactacctgagcacccagtccgccctgagcaaagaccccaacgagaagcgcgatcacatggtcctgctggagttcgtgaccgccgccgggatcactcacggcatggacgagctgtacaagtaa

### TCF12

#### 5′ Homologue sequence to pcDNA3.1-GFP-Flag-JunBΔIDR1-EPEA TCF12 coding sequence Homologue sequence to pcDNA3.1-GFP-Flag-JunBΔIDR1-EPEA3’

aggccgccgccaaggaacggatgaatccccagcaacaacgcatggccgctatagggaccgacaaggagctgagcgacctactggacttcagtgcgatgttttccccacctgttaatagtgggaaaactagaccaactacactgggaagcagtcaattcagtggatcaggtattgatgaaagaggaggtacaacatcttggggaacaagtggtcaaccaagtccttcctatgattcatctagaggttttacagacagccctcattacagtgatcacttgaatgacagtcgattaggagcccatgaaggcttgtccccaacacctttcatgaactcaaatctgatgggaaaaacatcagagagaggctcattttccctgtacagcagagatactggattaccaggctgtcaatctagtctcctgagacaagatctggggcttgggagcccagcacagctatcttcttcaggaaaacctgggacagcatactattcattctctgctacaagttccaggaggagaccactccatgactctgcagcgcttgatcccttgcaagcaaaaaaagtcagaaaggtgcctcctggtttgccttcttctgtatatgcaccatccccaaattcagatgatttcaaccgtgaatctcctagttatccatctcctaagccaccaaccagtatgttcgctagcactttctttatgcaagatgggacccacaattcttctgacctttggagttcatcaaatgggatgagccagcctggttttggtggaattctggggacctccacttcccacatgtctcaatccagtagttatggcaaccttcattcacatgaccgcttgagttatcctccacactcagtttcaccaacagacataaacacgagtcttccaccaatgtccagctttcatcgcggcagtaccagcagttcaccttacgttgctgcctcacacactcctcccatcaatggatcagacagcattctaggaaccagagggaatgctgctggaagctcacagacaggtgatgcacttggaaaggctttggcatctatttattctcctgaccataccagcagtagttttccgtcaaatccatcaacaccagttggatcaccttcacctctcacaggtaccagtcagtggccaagacctggagggcaagcaccttcatccccaagctatgaaaactcactccactccctgaaaaatcgagttgagcagcaacttcacgagcatttgcaagatgcaatgtccttcttaaaggatgtctgtgagcagtctcgaatggaggatcgtttagacagactggatgatgcaatccatgtgctgcggaaccatgctgtgggaccttccaccagtttgcctgctggtcacagtgatatacatagtttattgggaccatcccataatgcaccaattggaagcctcaattcaaactatggaggatcaagccttgttgcaagcagtcgatcagcttcaatggttggaactcatcgggaagactctgtcagtctcaatggcaatcattcagtcctgtctagtacagtcactacttcaagcacagacctgaaccataaaacacaagaaaattatagaggtggcttgcaaagtcagtctggaactgttgttacaacagaaatcaagactgaaaacaaagaaaaggatgaaaaccttcatgaacctccttcatcagatgacatgaagtcagatgatgaatcctcccaaaaagatatcaaggtttcatctagaggcagaacaagcagtactaatgaagatgaggatttgaaccctgaacagaagatagaaagggagaaggagaggcggatggctaacaatgccagagaacgcttacgcgtgcgggatattaatgaagcattcaaagagcttggccgaatgtgtcagcttcacttgaagagtgaaaaaccccaaacaaaactccttattcttcatcaagccgtggcagtcatccttagtctagaacagcaagtcagagagaggaaccttaaccccaaagcagcctgccttaagagaagggaagaagaaaaagtttctgccgtatcggcagagccgccaaccacactgccaggaacccatcctgggcttagtgaaactaccaaccctatgggtcatatgtaactcgagtctagagggcccgt

## Supporting information







## Abbreviations used:

bHLH: basic helix-loop-helix
DB: DNA-binding
IDR: intrinsically disordered region
IF: immunofluorescence
IFI16: interferon alpha-inducible protein 6
JUNB: transcription factor JunB
TCF12: transcription factor 12
TEAD: TEA domain
TFs: transcription factors
YAP: Yes-associated protein.
