# Supplementary material for: Investigating the functional components of YAP condensates
Source: Mol Biol Cell. 2026 May 13;37(7):br20. doi: 10.1091/mbc.E25-11-0532 (PMC13242282; doi:10.1091/mbc.E25-11-0532)
Supplement: Supplementary file 1 [file mbc-37-br20-s001.pdf]

Supplemental Materials

Molecular Biology of the Cell

Bellot *et al*.

Figure S1

A)

Biological Process (10 min)		
Term description	observed Peptide count	Fold Enrichment
Regulation of mRNA catabolic process	5	1.17
mRNA splicing via spliceosome	6	1.1
RNA splicing	8	1.09
Regulation of mRNA metabolic process	6	1.04
mRNA processing	7	0.96
Regulation of RNA metabolic process	18	0.47
Regulation of gene expression	20	0.4

Biological Process (2 h)		
Term description	observed Peptide count	Fold Enrichment
Regulation of RNA splicing	5	1.23
Regulation of mRNA stability	5	1.15
RNA splicing	10	1.11
mRNA splicing via spliceosome	7	1.09
mRNA processing	9	1
mRNA metabolic process	10	0.88
RNA metabolic process	13	0.63
Nucleic acid metabolic process	16	0.58
Gene expression	14	0.54
Heterocycle metabolic process	18	0.51
Organic cyclic compound metabolic process	18	0.47
Cellular nitrogen compound metabolic process	18	0.45

Figure S2

A)

Biotinylated proteins and potential functions				
**Protein**	**Potential functions**	**no sorb**	**10 min sorb**	**2 h sorb**
**AFAP1**	Actin filament-associated protein 1			√
**CACYBP**	Participates in the ubiquitin-mediated degradation of beta-catenin (CTNNB1); Tumor progression.			√
**DDX46**	mRNA splicing via spliceosome	√	√	√
**FGFR3**	Cell proliferation, differentiation and apoptosis			√
**GPATCH4**	nucleoli and Cajal bodies; regulate cell growth and nucleolar structure	√	√	√
**HELLS**	silencing of the imprinted CDKN1C gene through DNA methylation	√	√	√
**HN1L**	Affinity Capture-MS			√
**HNRNPF**	mRNA splicing via spliceosome	√	√	√
**HNRNPU**	mRNA splicing via spliceosome			√
**IFI16**	transcription regulation (TP53)	√	√	√
**JUNB**	transcription coregulator activity	√	√	√
**MED23**	transcription coregulator activity	√		√
**NAB1**	transcription coregulator activity		√	√
**NONO**	mRNA splicing via spliceosome	√	√	√
**ORC3**	DNA replication			√
**PUF60**	mRNA splicing via spliceosome		√	
**RBM14**	transcription coregulator activity		√	√
**RPL9**	L ribosomal proteins			√
**SART1**	mRNA splicing via spliceosome	√	√	√
**SERBP1**	regulation of mRNA catabolic		√	√
**SF3B1**	mRNA splicing via spliceosome		√	√
**SUGP2**	mRNA processing		√	√
**TACC2**	Plays a role in the microtubule-dependent coupling of the nucleus and the centrosome	√	√	√
**TAF15**	regulation of mRNA catabolic; transcription coregulator activity		√	√
**TCF12**	TF		√	
**THRAP3**	transcription coregulator activity			√
**UBAP2L**	Required for efficient formation of stress granules		√	√
**VIM**	regulation of mRNA catabolic	√	√	√
**ZC3H14**	regulation of mRNA catabolic		√	
**ZC3HAV1**	regulation of mRNA catabolic		√	
**ZFP91**	activate MAP3K14/NIK; E3 ubiquitin-protein ligase			√
**ZMAT2**	mRNA splicing via spliceosome			√
**ZNF280D**	TF			√
**ZNF281**	Transcription repressor		√	

Figure S3

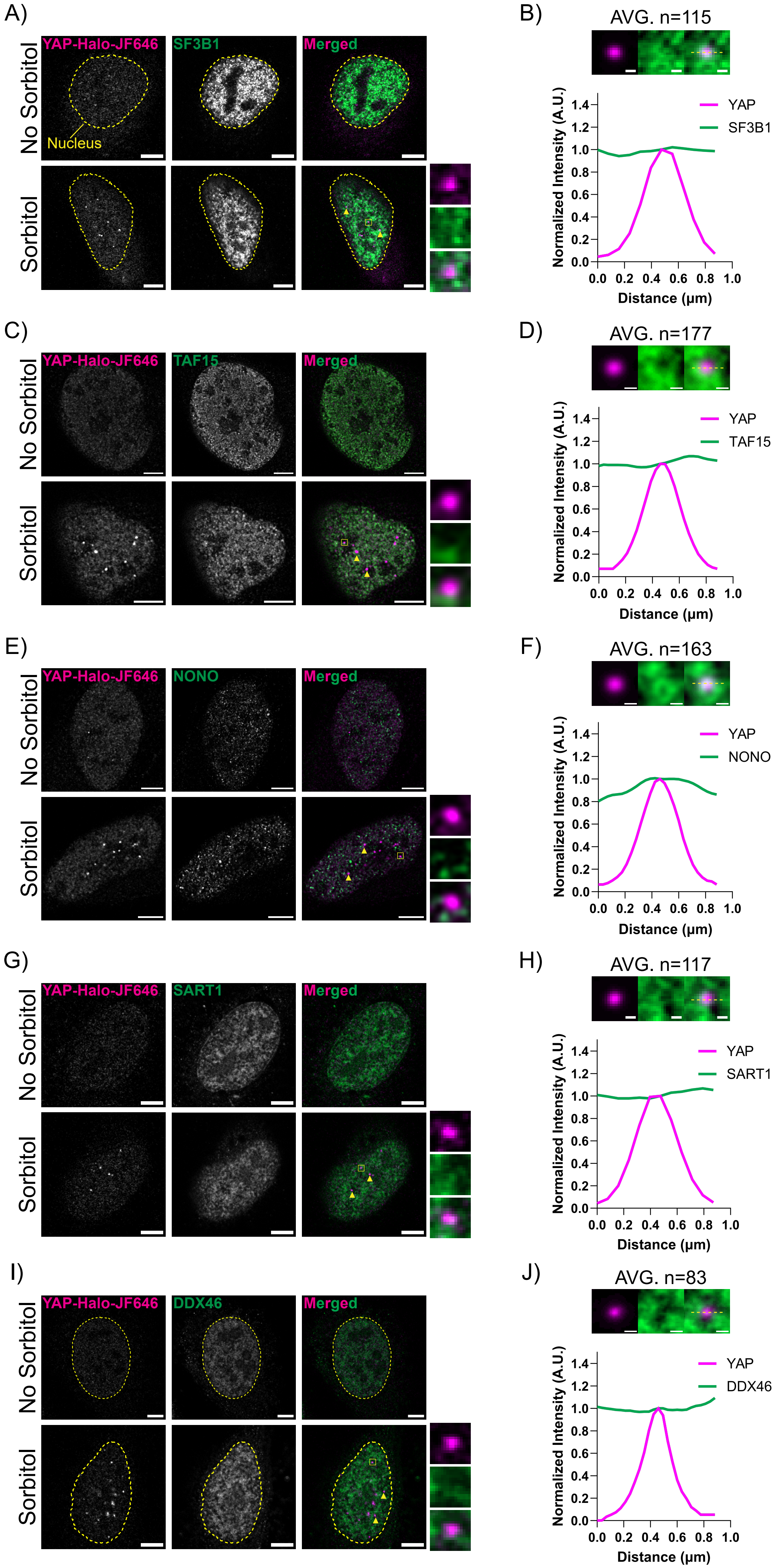
A)
YAP-Halo-JF646
SF3B1
Merged
No Sorbitol
Sorbitol
Nucleus
B)
AVG. n=115
Normalized Intensity (A.U.)
Distance (µm)
YAP
SF3B1
C)
YAP-Halo-JF646
TAF15
Merged
No Sorbitol
Sorbitol
D)
AVG. n=177
Normalized Intensity (A.U.)
Distance (µm)
YAP
TAF15
E)
YAP-Halo-JF646
NONO
Merged
No Sorbitol
Sorbitol
F)
AVG. n=163
Normalized Intensity (A.U.)
Distance (µm)
YAP
NONO
G)
YAP-Halo-JF646
SART1
Merged
No Sorbitol
Sorbitol
H)
AVG. n=117
Normalized Intensity (A.U.)
Distance (µm)
YAP
SART1
I)
YAP-Halo-JF646
DDX46
Merged
No Sorbitol
Sorbitol
J)
AVG. n=83
Normalized Intensity (A.U.)
Distance (µm)
YAP
DDX46

Figure S4

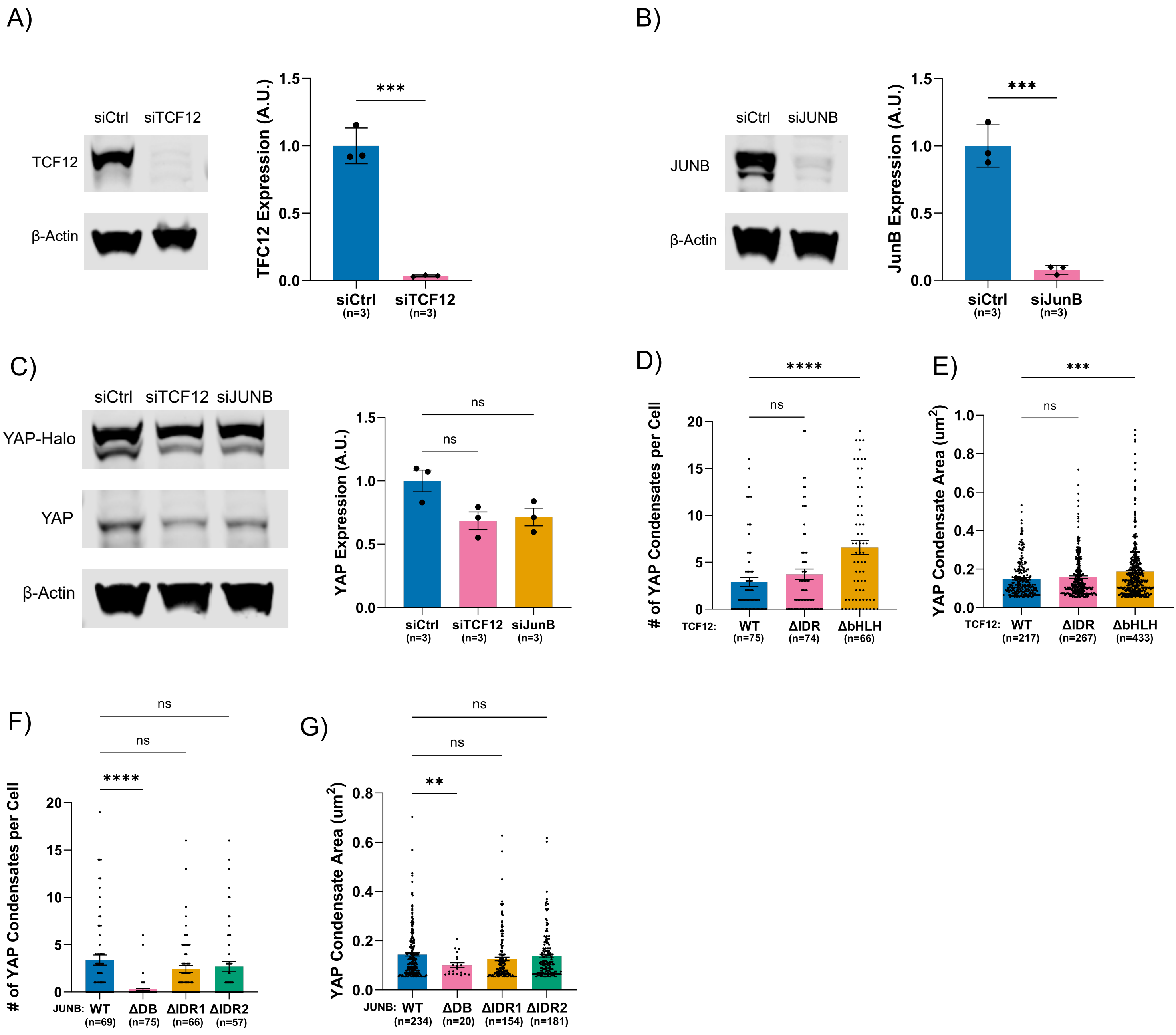
A)
siCtrl siTCF12
TCF12
β-Actin
TFC12 Expression (A.U.)
***
siCtrl (n=3)
siTCF12 (n=3)
B)
siCtrl siJUNB
JUNB
β-Actin
JunB Expression (A.U.)
***
siCtrl (n=3)
siJunB (n=3)
C)
siCtrl siTCF12 siJUNB
YAP-Halo
YAP
β-Actin
YAP Expression (A.U.)
ns
siCtrl (n=3)
siTCF12 (n=3)
siJunB (n=3)
D)
# of YAP Condensates per Cell
****
ns
TCF12: WT (n=75) ΔIDR (n=74) ΔbHLH (n=66)
E)
YAP Condensate Area (um^2)
***
ns
TCF12: WT (n=217) ΔIDR (n=267) ΔbHLH (n=433)
F)
# of YAP Condensates per Cell
ns
****
JUNB: WT (n=69) ΔDB (n=75) ΔIDR1 (n=66) ΔIDR2 (n=57)
G)
YAP Condensate Area (um^2)
ns
**
JUNB: WT (n=234) ΔDB (n=20) ΔIDR1 (n=154) ΔIDR2 (n=181)

Original uncropped WB membrane of Fig 4 S4 A, B, C

A) Related to Fig S4 A, B

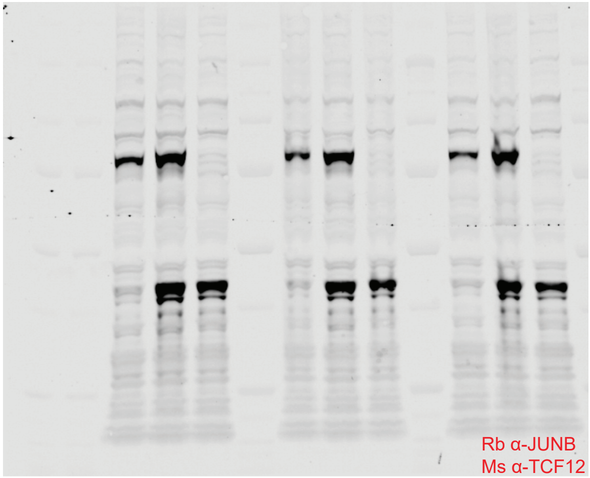

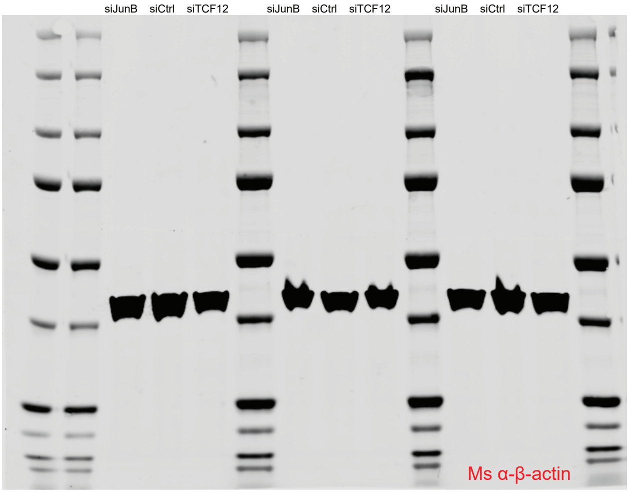

B) Related to Fig S4 C

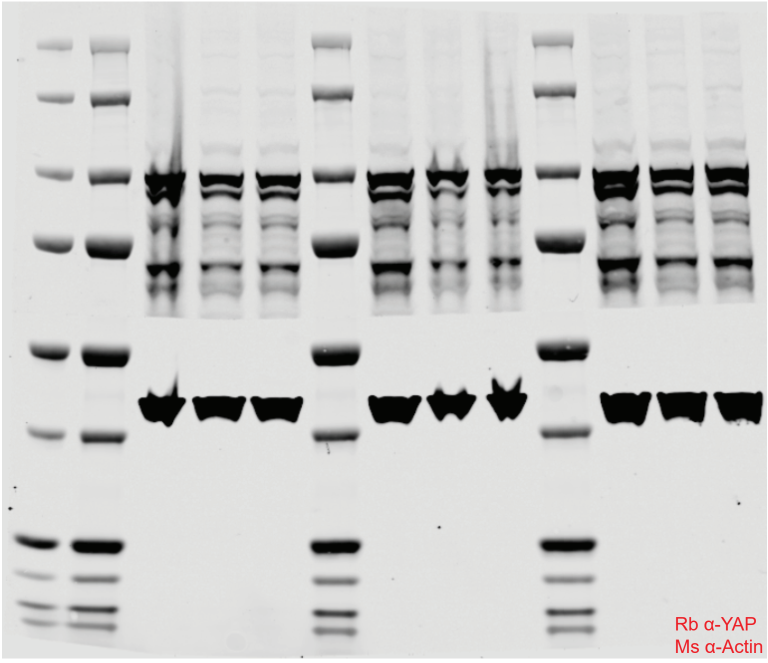

Original uncropped WB membrane of Fig 5 B, C and other two related biological replicates

A) Related to Fig 5 B, C

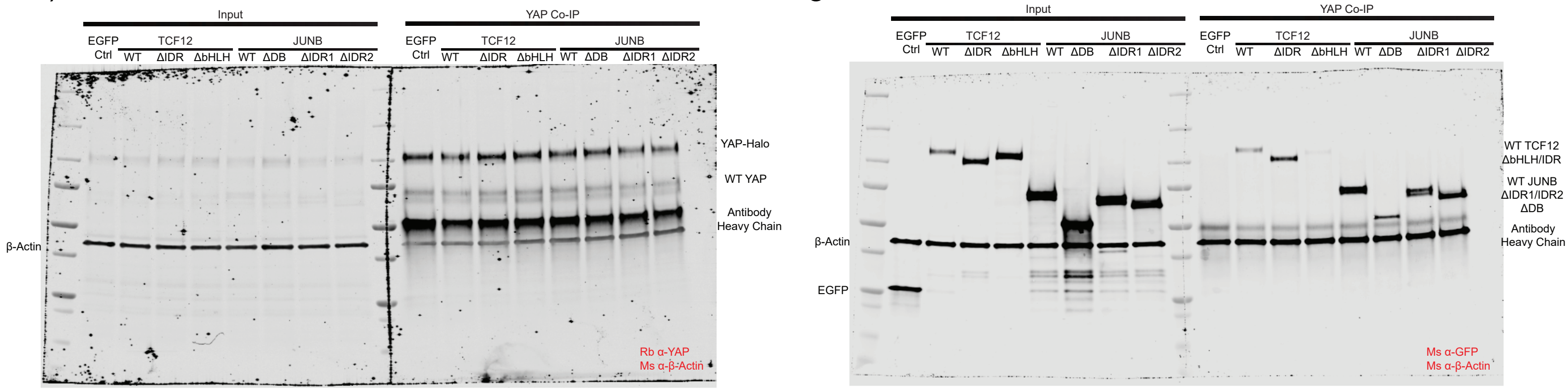

B) Additional biological replicate related to Fig 5 B, C

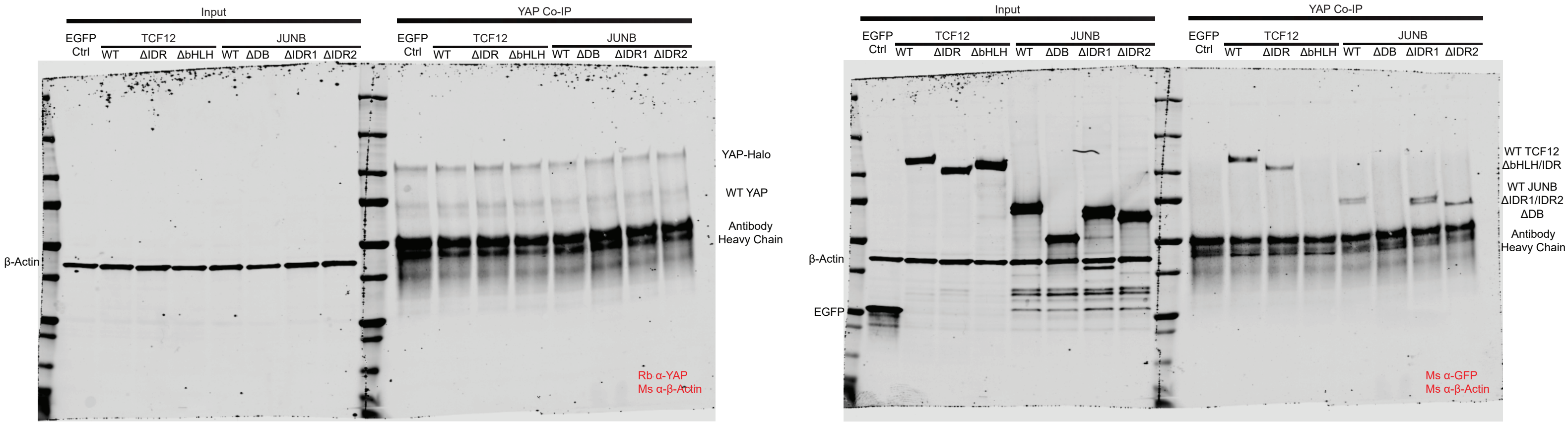

C) Additional biological replicate related to Fig 5 B, C

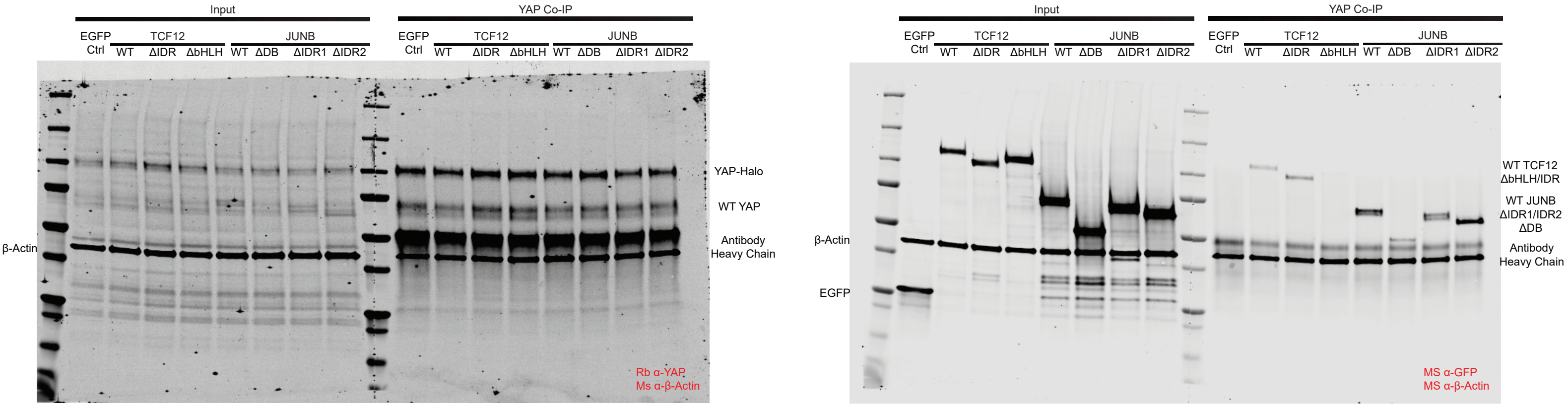

Supplemental Figure S1. GO enrichment analysis of BP terms for proteins biotinylated by miniTurbo-YAP-EGFP in HeLa cells treated with 0.2 M sorbitol for 10 minutes and 2 h respectively.

Supplemental Figure S2. Proteins and their potential function identified by mini-Turbo in HeLa cells treated with no sorbitol, 0.2 M sorbitol for 10 minutes and 2 h respectively.

Supplemental Figure S3. Colocalization analysis of candidate proteins with endogenous YAP condensates. (A) Representative immunofluorescence images of U-2 OS YAP-HaloTag cells, with JF646 Halo Dye and SF3B1 antibody staining, after control (no sorbitol) and 5 min 0.2 M sorbitol treatments. Insets show intensity of SF3B1 around a YAP condensate. (B) Averaged (AVG) images centering on individual YAP condensates (magenta) formed after 5 min 0.2M sorbitol treatment, showing no enrichment of SF3B1 signal (green) in the center. Line plots (below) show normalized intensity of indicated channels along a white dotted line drawn through the merged averaged image (above). (C-J) Similar to (A-B) but show no enrichment of TAF15 (C-D), NONO (E-F), SART1 (G-H) and DDX46 (I-J) in YAP condensates formed after 5 min 0.2M sorbitol treatments. In (A, C, E, G, I), scale bars = 5 µm, scale bars = 0.3 µm for inset; (B, D, F, H, J), scale bars = 0.3 µm. Arrows denote a few YAP condensates. Insets are magnified views of the boxed region. Yellow dashed lines denote nuclear boundaries.

Supplemental Figure S4. (A) Western blot validation of TCF12 knockdown by siRNA. Left: representative blots from three independent experiments. Right: quantification of relative TCF12 band intensity normalized to β-actin. (B) Western blot validation of JUNB knockdown by siRNA. Left: representative blots from three independent experiments. Right: quantification of relative JUNB band intensity normalized to β-actin. (C) Western blot analysis of WT YAP and YAP-Halo expression following TCF12 or JUNB knockdown, compared with siCtrl. Left: representative blots from three independent experiments. Right: quantification of total YAP band intensity, calculated by combining WT YAP and YAP-Halo signals and normalizing to β-actin. (D-G) Quantification of average YAP condensate number (D, F) and average size (E, G) on U-2 OS YAP-HaloTag cells transfected with (D, E) GFP-tagged WT TCF12 and mutants, as well as, (F, G) GFP-tagged WT-JUNB and mutants, formed after 5 min 0.2 M sorbitol treatments. Data are shown as mean ± SEM; Statistical significance was assessed using unpaired t-test. ** $p<0.01$; ***$p<0.001$, **** $p < 0.0001$.
