# Supplementary material for: Investigating the functional components of YAP condensates
Source: Mol Biol Cell. 2026 May 13;37(7):br20. doi: 10.1091/mbc.E25-11-0532 (PMC13242282; doi:10.1091/mbc.E25-11-0532)
Supplement: Supplementary file 3 [file reviewer_reportE25-11-0532.pdf]

Investigating the functional components of YAP condensates

Janelle Bellot, Siyuan Hao, Yihuan Li, and Danfeng Cai

Corresponding author(s): Danfeng Cai, Johns Hopkins University

Review Timeline:

Submission Date:	2025-11-04
Editorial Decision:	2025-12-02
Revision Received:	2026-04-17
Accepted:	2026-04-30

Editor-in-Chief: John Aitchison

Transaction Report:

(Note: With the exception of the correction of typographical or spelling errors that could be a source of ambiguity, letters and reports are not edited. The original formatting of letters and referee reports may not be reflected in this compilation.)

RE: Manuscript #E25-11-0532
TITLE: Investigating the functional components of YAP condensates

Dear Dr. Cai:

Both reviewers appreciate the significance of your work, but do think that TCF12 and JUNB's roles in YAP condensate formation and their functional relevance need to be better supported. I therefore invite you revise the manuscript.

Sincerely,

Huaiying Zhang
Monitoring Editor
Molecular Biology of the Cell

Dear Dr. Cai,

The review of your manuscript, referenced above, is now complete. The Monitoring Editor has decided that your manuscript is not acceptable for publication at this time, but may be deemed acceptable after specific revisions are made, as described in the Monitoring Editor's decision letter above and the reviewer comments below.

A reminder: Please do not contact the Monitoring Editor directly regarding your manuscript. If you have any questions regarding the review process or the decision, please contact the MBoC Editorial Office (mboc@ascb.org).

When submitting your revision include a rebuttal letter that details, point-by-point, how the Monitoring Editor's and reviewers' comments have been addressed. (The file type for this letter must be "rebuttal letter"; do not include your response to the Monitoring Editor and reviewers in a "cover letter.") Please bear in mind that your rebuttal letter will be published with your paper if it is accepted, unless you have opted out of publishing the review history.

Authors are allowed 90 days to submit a revision. If this time period is inadequate, please contact us at mboc@ascb.org.

Revised manuscripts are assigned to the original Monitoring Editor whenever possible. However, special circumstances may preclude this. Also, revised manuscripts are often sent out for re-review, usually to the original reviewers when possible. The Monitoring Editor may solicit additional reviews if it is deemed necessary to render a completely informed decision.

In preparing your revised manuscript, please follow the instruction in the Information for Authors (www.molbiolcell.org/info-for-authors). In particular, to prepare for the possible acceptance of your revised manuscript, submit final, publication-quality figures with your revision as described.

To submit the rebuttal letter, revised manuscript, and figures, use this link: Link Not Available

MBoC offers the option to publish your work with immediate open access. Open access can increase the discoverability and usability of your research. If you would like to publish your paper with immediate open access but did not select this option during initial submission, please contact the editorial office at mbc@ascb.org.

Authors of Articles and Brief Communications are also encouraged to create a short video abstract to accompany their article when it is published. These video abstracts, known as Science Sketches, are up to 2 minutes long and will be published on YouTube and then embedded in the article abstract. Science Sketch Editors on the MBoC Editorial Board will guide you as you prepare your video. Information about how to prepare and submit a video abstract is available at www.molbiolcell.org/science-sketches. Please contact mboc@ascb.org if you are interested in creating a Science Sketch.

Sign up for MBoC's new content alerts here https://tinyurl.com/3bcsyk3e

Thank you for submitting your manuscript to MBoC. We look forward to receiving your revised paper.

Sincerely,

Eric Baker
Journal Production Manager
MBoC Editorial Office

mbc@ascb.org

Reviewer #1 (Remarks to the Author):

The manuscript "Investigating the functional components of YAP condensates" by Bellot et al. addresses an interesting and important question about the molecular composition and functional regulation of YAP condensates under hyperosmotic stress. The study identifies JUNB and TCF12 as novel YAP condensate components and demonstrates their requirement for YAP condensate formation. The topic is timely and relevant to the field of Hippo signaling and phase separation biology. However, several key points need to be addressed to strengthen the mechanistic depth and rigor of the study.
Major Concerns
1. Lack of ChIP-seq or chromatin occupancy evidence for YAP/TCF12/JUNB co-localization.
While the authors identify TCF12 and JUNB as novel YAP condensate components and show their co-enrichment by immunofluorescence, there is no evidence demonstrating that these factors co-occupy chromatin or regulate common genomic loci. ChIP-seq or CUT&Run experiments for YAP, TCF12, and JUNB under hyperosmotic stress would be crucial to support the proposed co-regulatory mechanism and to link condensate formation to transcriptional activation.
2. Quantitative analysis of YAP condensate morphology (Figure 3 and 4).
The YAP condensates presented in Figure 3 and 4 appear not very comparable in size to those previously reported in Cai et al. (2019, Nat Cell Biol) and the recent work by Yaguang et al. (Mol Cell, 2024), which may be caused by different cell types or different lab environment. Does YAP form different sizes of condensates in different cell types? Quantitative comparisons of condensate dimensions and fluorescence intensity relative to previously published benchmarks are necessary to substantiate novelty. Or some discussion on the difference may be helpful.
3. Functional link to Hippo target genes.
In Figure 4, knockdown of TCF12 and JUNB affects YAP condensate formation and reduces Cyr61 expression, but the authors only test two target genes (Cyr61 and CTGF). To strengthen the conclusion that TCF12 and JUNB regulate YAP transcriptional output, the authors should measure additional canonical Hippo/YAP target genes (e.g., ANKRD1, AMOTL2, BIRC5, etc.) following knockdown. This would clarify whether the effects are specific to certain genes or reflect a broader transcriptional impairment of YAP activity.

Minor Comments

• Include quantification (mean {plus minus} SD, n values) for all immunofluorescence-based analyses in the main figures.
• Ensure consistent use of gene/protein symbols (e.g., CTGF vs Ctgf) throughout the manuscript.

Reviewer #2 (Remarks to the Author):

The manuscript by Bellot et al. employs proximity-based proteomics to identify novel components of YAP condensates, specifically under hyperosmotic stress. The authors identify TCF12 and JUNB as enriched components and demonstrate that their depletion prevents YAP condensate formation and inhibits transcription of the target gene Cyr61.

While the identification of new interactors using an advanced technique (miniTurbo proximity labeling) is a solid starting point, the study is unfortunately halted by a critical lack of mechanistic depth, inconsistent functional data, and insufficient experimental controls. The conclusions drawn are largely correlative and do not establish a robust molecular mechanism for how TCF12 or JUNB regulate YAP phase separation. This work, as currently presented, is premature for publication.
Major comments: 1. Absence of Essential Controls for Proximity Proteomics. The stringency and reliability of the mass spectrometry data are compromised by critical missing controls for the miniTurbo assay: Missing Biotin Background Control: The authors utilize HeLa cells without the miniTurbo-YAP construct (WT) as a negative control to account for endogenous biotinylation. However, a key control is the transfected cell line (miniTurbo-YAP-EGFP-expressing cells) incubated without exogenous biotin. This control is essential to rule out artifactual labeling caused by non-specific streptavidin pull-down of highly abundant proteins or construct background binding independent of the biotinylation reaction. Without this, the background noise cannot be accurately defined. Small and Biologically Unexplained Proteome: The number of identified proteins (23 in control, 33 at 10 min, 39 at 2 h) 6 is strikingly low for proximity labeling. This limited output weakens confidence in the comprehensiveness of the dataset targeting a critical transcriptional co-activator. 2. Contradictory Functional Requirement for Transcription. The core finding regarding function is inconsistent, severely questioning the requirement of TCF12/JUNB-dependent condensates for YAP activity: The depletion of TCF12 or JUNB abolishes condensate formation and decreases the expression of the YAP target gene Cyr61. However, the expression of another canonical YAP target gene CTGF, is reported as not significantly affected (ns) upon knockdown of either TCF12 or JUNB (Figure S3C, D).This is a major contradiction. If the TCF12/JUNB-dependent condensates are required for YAP transcriptional activation, then all downstream targets should be suppressed upon condensate abolition. The fact that CTGF remains unaffected suggests either that these condensates are dispensable for a subset of YAP function, or

that the observed effect on Cyr61 is specific to a TCF12/JUNB regulatory mechanism independent of phase separation. This major inconsistency needs to be resolved through further functional data and a comprehensive discussion. 3. Lack of Mechanistic and Biophysical Characterization. The manuscript provides no data to explain how TCF12 and JUNB influence phase separation. Missing Biophysical Data (FRAP): Phase separation is a biophysical process. Showing that TCF12/JUNB depletion prevents condensate formation (Figure 4) is purely correlative. The authors must perform Fluorescence Recovery After Photobleaching (FRAP) experiments to measure the exchange dynamics, mobility, and liquid-like properties of YAP condensates before and after knockdown. Do these proteins serve as scaffolds? Do they change the viscosity or maturation rate? Structural Basis and Scaffolding: There is no attempt to map the domains responsible for the interaction or nucleation. The manuscript claims TCF12 and JUNB are "required for YAP condensate formation", implying a scaffolding or nucleation role. The authors should investigate if they bind YAP directly and if they have unique low-complexity domains (IDRs) that facilitate multivalent interactions. 4. Superficial Analysis of Proteomics Data Over TimeThe analysis of the transition from 10 minutes to 2 hours post-sorbitol is descriptive and lacks biological interpretation:GO Term Interpretation (Figure 2E): The authors note the distinct clustering of proteins at 10 min and 2 hrs. The Gene Ontology (GO) term "Nucleic acid metabolic process" shows greater enrichment at 2 hours compared to 10 minutes (Figure 2E). Given the biological model-enhancer clustering (10 min) shifting to active transcription (2 h) -the authors need to discuss which specific nucleic acid metabolic proteins are enriched and propose a mechanism for their sequential recruitment in relation to the transcriptional activation phase. Simply presenting a bar plot of GO terms is insufficient for a research article. Minor Points 1. Data Presentation and Visualization, Error Bar Clarity: The legend for Figure 4 states that error bars represent mean SD, while the legend for Figure 1 and others states mean SEM. This discrepancy must be corrected for statistical consistency throughout the paper.YAP Quantification Context: In Figure 4G, the Western blot and subsequent quantification show the effect of TCF12/JUNB knockdown on both YAP-Halo and endogenous YAP. Please ensure the quantification clearly specifies how the signals from the exogenous YAP-HaloTag fusion protein and the endogenous wild-type YAP protein are integrated or separated, particularly since the siRNA targets the endogenous system. Localization of IFI16 (Figure 3G): IFI16 appears to be concentrated heavily around the nuclear periphery in the "No Sorbitol" condition. This known shuttling or peripheral localization should be noted in the text, as it adds context to its subsequent enrichment inside the nuclear condensates upon sorbitol treatment. 2. Technical and Terminological Clarity Missing Primer Information: While primers for CTGF are listed in the Materials and Methods, the primers used for Cyr61 (quantified in Figure 4H, I) should also be provided for full reproducibility.Clarity on IFI16 Necessity: The authors state they could not test the necessity of IFI16 because 3 days of knockdown affected cell viability. This should be stated clearly as an experimental limitation in the main text. Protein Exclusions: The authors should briefly clarify the selection criteria used to narrow the list of 33 identified proteins at 10 min (Figure 2A) down to the handful of candidates selected for IF validation (Figures 3 and S2).

Reviewer #1 (Remarks to the Author):

The manuscript "Investigating the functional components of YAP condensates" by Bellot et al. addresses an interesting and important question about the molecular composition and functional regulation of YAP condensates under hyperosmotic stress. The study identifies JUNB and TCF12 as novel YAP condensate components and demonstrates their requirement for YAP condensate formation. The topic is timely and relevant to the field of Hippo signaling and phase separation biology. However, several key points need to be addressed to strengthen the mechanistic depth and rigor of the study.

We would like to thank the reviewer for the overall positive comments. We have now revised the manuscript to incorporate most of the suggested changes, and hope the paper is now ready for acceptance.

Major Concerns

1. Lack of ChIP-seq or chromatin occupancy evidence for YAP/TCF12/JUNB co-localization. While the authors identify TCF12 and JUNB as novel YAP condensate components and show their co-enrichment by immunofluorescence, there is no evidence demonstrating that these factors co-occupy chromatin or regulate common genomic loci. ChIP-seq or CUT&Run experiments for YAP, TCF12, and JUNB under hyperosmotic stress would be crucial to support the proposed co-regulatory mechanism and to link condensate formation to transcriptional activation.

We really appreciate the important suggestion from the reviewer. However, the limited timeframe for paper revision prevents us from performing the suggested ChIP-seq or CUT&RUN experiments and analysis in time. Despite this, it has been shown that YAP/TAZ/TEAD and AP-1 (containing JUNB) co-bind at enhancers to drive oncogenic growth (Zanconato et al., 2015). Our new JUNB domain deletion data showed that the DNA-binding (DB) domain of JUNB was important for direct binding with YAP. Without DB (JUNΔDB), JUNB does not bind YAP, and YAP condensates do not form. This intriguing new data suggests that YAP condensates form at JUNB loci through direct interactions of YAP with DNA-bound JUNB (**updated Figure 5C, D, K, and N; reviewer Figure 1.1 B-E below**). On the other hand, deleting the DNA-binding bHLH domain of TCF12 (TCF12ΔbHLH) abrogated direct TCF-YAP binding but increased the incorporation of TCF12 into YAP condensates, indicating that YAP condensates do not form at TCF12-bound DNA sites (**updated Figure 5B, D, H and I; reviewer Figure 1.1 A, C, F, G below**). These results collectively indicate that YAP condensates form at JUNB loci. Thank you again for the question that prompted our follow-up experiments, leading to this important insight.

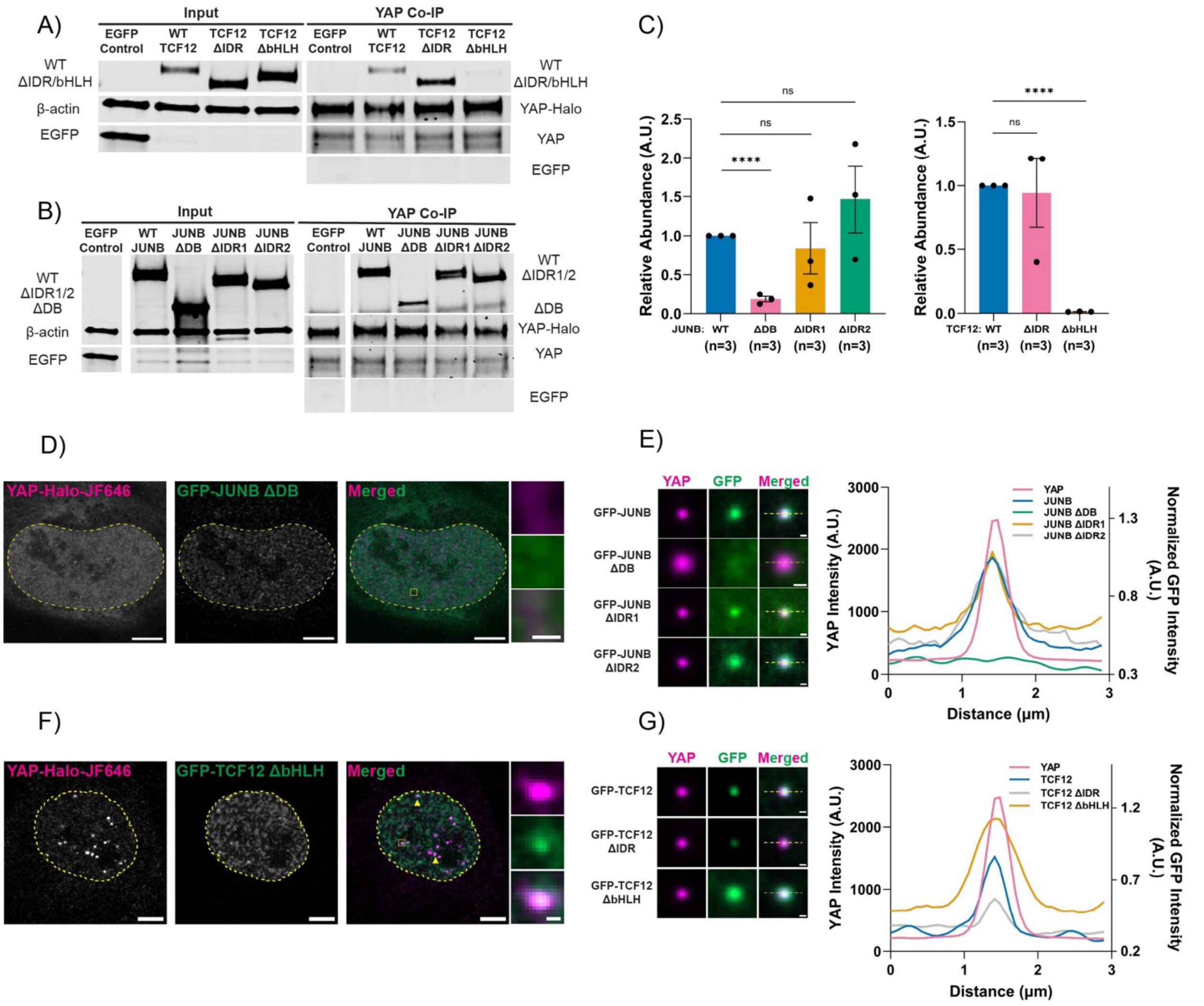

Reviewer Fig 1.1. Specific domains of JUNB and TCF12 are important for direct YAP binding and incorporation into YAP condensates. (A-B) Co-immunoprecipitation assays demonstrate YAP interactions with (A) TCF12 and TCF12 mutants as well as (B) JUNB and JUNB mutants. (C) Quantifications of proteins abundance for mutants of TCF12 (left) and JUNB (right) in the YAP Co-IP fraction relative to WT TCF12 or JUNB. (D) Representative immunofluorescence images of U-2 OS YAP-HaloTag cells stained with JF646 Halo Dye, and transfected with GFP-JUNBΔDB, after 5 min 0.2 M sorbitol treatments. Arrows denote a few YAP condensates. Insets are magnified views of the boxed region. Yellow dashed lines denote nuclear boundaries. (E) Averaged images centering on individual YAP condensate (magenta) formed after 5 min 0.2M sorbitol treatment and localization of GFP-tagged WT JUNB, as well as mutants relative to YAP condensate (left) Line plots (right) show raw YAP intensity and GFP intensity normalized to GFP control along the yellow dotted line drawn through the merged averaged image (n=234 for JUNB, n=20 for JUNBΔDB, n=161 for JUNBΔIDR1, n=154 for JUNBΔIDR2). (F) Similar to D but show U-2 OS YAP-HaloTag cells transfected with GFP-TCF12ΔbHLH. (G) Similar to E, but show GFP-tagged WT TCF12, and mutants (n=217 for TCF12, n=267 for TCF12ΔIDR, n=433 for TCF12ΔbHLH). (D, F) scale bars = 5 μm, scale bars = 0.3 μm for inset; (B, D, F, H, J), scale bars = 0.3 μm; (E, G) scale bars = 0.3 μm. Statistical significance was assessed using t-test ns, not significant; **** $p < 0.0001$. Data are shown as

Mean ± SEM.

2. Quantitative analysis of YAP condensate morphology (Figure 3 and 4).
The YAP condensates presented in Figure 3 and 4 appear not very comparable in size to those previously reported in Cai et al. (2019, Nat Cell Biol) and the recent work by Yaguang et al. (Mol Cell, 2024), which may be caused by different cell types or different lab environment. Does YAP form different sizes of condensates in different cell types? Quantitative comparisons of condensate dimensions and fluorescence intensity relative to previously published benchmarks are necessary to substantiate novelty. Or some discussion on the difference may be helpful.

We appreciate the important question from the reviewer. Following the suggestions, we have quantified the YAP condensate size in the current study, and compared it to the endogenous YAP condensate size in the same U-2 OS YAP-HaloTag cell line reported in Hao et al., iScience 2024 (**Reviewer Fig. 1.2 below**). The results showed comparable sizes of YAP condensates. The slight discrepancy may be due to different intensity thresholds used in different studies. The intensity of YAP condensates is not comparable across studies due to the different laser and detector gain settings employed by different scientists. Thank you for your understanding.

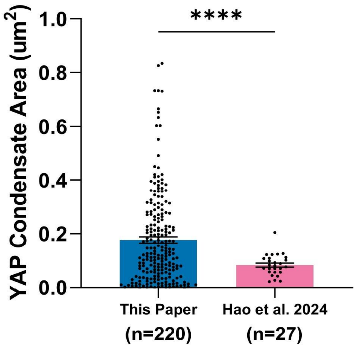

Reviewer Fig 1.2. Quantifications of area of YAP condensates formed in U-2 OS YAP-HaloTag cells in this study compared to Hao *et al.* 2024. Statistical significance was assessed using unpaired t-test ; **** $p < 0.0001$. Error bars: Mean ± SEM.

3. Functional link to Hippo target genes.
In Figure 4, knockdown of TCF12 and JUNB affects YAP condensate formation and reduces Cyr61 expression, but the authors only test two target genes (Cyr61 and CTGF). To strengthen the conclusion that TCF12 and JUNB regulate YAP transcriptional output, the authors should measure additional canonical Hippo/YAP target genes (e.g., ANKRD1, AMOTL2, BIRC5, etc.) following knockdown. This would clarify whether the effects are specific to certain genes or reflect a broader transcriptional impairment of YAP activity.

We appreciate the comment. Following the suggestion, we have now assayed additional Hippo pathway/ YAP target genes, including *BIRC5* and *AMOTL2* (**updated Figure 4H,I; reviewer Fig. 1.3 below**). Our data shows that knocking down TCF12 and JUNB decreases the expression of these target genes, reflecting a broader transcriptional impairment of YAP activity. On the other hand, we speculate that the lack of effects of knocking down TCF12 and JUNB on *CTGF* expression (**Figure 4J**) reflects that CTGF may be regulated by YAP outside of

condensates. Therefore, we added the following discussion to the text: “The level of *CTGF*, however, is not affected after TCF12 or JUNB k.d. possibly because *CTGF* expression is mainly driven by YAP outside of condensates in the U-2 OS cells”. Thank you again for the suggestions.

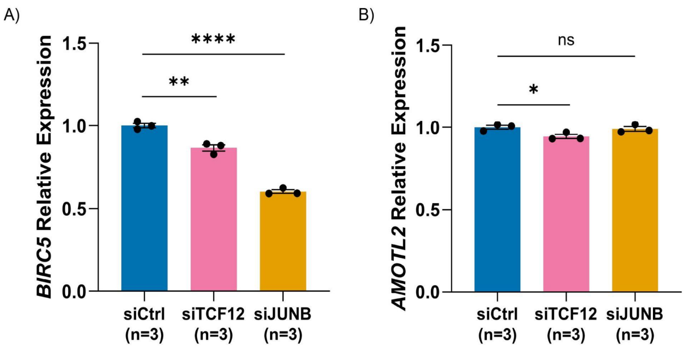

Reviewer Fig 1.3. TCF12 and JUNB regulated YAP target gene expression. Expression levels of YAP target genes (A) BIRC5, (B) AMOTL2, were quantified by RT-qPCR from siCtrl versus siTCF12 and siJUNB treated cells. Statistical significance was assessed using unpaired t-test; ns, not significant; * $p<0.05$; ** $p<0.01$; **** $p < 0.0001$. Error bars: Mean ± SEM.

Minor Comments

• Include quantification (mean +- SD, n values) for all immunofluorescence-based analyses in the main figures.

We appreciate the comments. The original submission included mean +- SEM/SD for all immunofluorescence-based analyses. We have now added the n values for all quantifications. Thanks again for the feedback.

• Ensure consistent use of gene/protein symbols (e.g., CTGF vs Ctgf) throughout the manuscript.

We have fixed the issue to consistently write the target genes as *CTGF* etc. Thanks again for the suggestion.

Reviewer #2 (Remarks to the Author):

The manuscript by Bellot et al. employs proximity-based proteomics to identify novel components of YAP condensates, specifically under hyperosmotic stress. The authors identify TCF12 and JUNB as enriched components and demonstrate that their depletion prevents YAP condensate formation and inhibits transcription of the target gene Cyr61.

While the identification of new interactors using an advanced technique (miniTurbo proximity labeling) is a solid starting point, the study is unfortunately halted by a critical lack of mechanistic depth, inconsistent functional data, and insufficient experimental controls. The conclusions drawn are largely correlative and do not establish a robust molecular mechanism

for how TCF12 or JUNB regulate YAP phase separation. This work, as currently presented, is premature for publication.

We appreciate the reviewer's thoughtful comments. In response to these suggestions, we have incorporated key experiments to further investigate the molecular mechanisms by which TCF12 and JUNB regulate YAP condensate formation. We have also expanded our analysis to include additional YAP target genes, allowing for a more comprehensive assessment of how TCF12 and JUNB influence YAP transcriptional activity. We hope that these revisions have strengthened the manuscript and that it is now suitable for publication in *Molecular Biology of the Cell*.

Major comments:
1. Absence of Essential Controls for Proximity Proteomics. The stringency and reliability of the mass spectrometry data are compromised by critical missing controls for the miniTurbo assay: Missing Biotin Background Control: The authors utilize HeLa cells without the miniTurbo-YAP construct (WT) as a negative control to account for endogenous biotinylation. However, a key control is the transfected cell line (miniTurbo-YAP-EGFP-expressing cells) incubated without exogenous biotin. This control is essential to rule out artifactual labeling caused by non-specific streptavidin pull-down of highly abundant proteins or construct background binding independent of the biotinylation reaction. Without this, the background noise cannot be accurately defined.

We appreciate the expert advice from the reviewer. We would like to politely ask for the omission of this control due to the limited timeframe for paper revision, as including it would require us to redo the entire TurboID experiment. We are confident that not including the said control would not affect our overall conclusion, since:

1. Our study focuses on the differential interactome of YAP before and after sorbitol treatments. Since the 'background' (non-specific binding or endogenous biotinylation) is constant across both treated and untreated miniTurbo-YAP lines, these artifacts are subtracted during the differential enrichment analysis, ensuring that the reported hits are YAP condensate - specific interactors.
2. In the final list of proteins (**Supplemental Table 1**), we only included the ones with confirmed biotinylation, effectively eliminating the contributions from non-biotinylated highly abundant proteins or miniTurbo-YAP-EGFP stuck on the beads.
3. Most research papers that perform proximity-based ligation proteomics do not include the no biotin control but instead use other biological controls (Larochelle *et al.*, 2019; Zhang *et al.*, 2019; Su *et al.*, 2023), including the original Alice Ting lab paper that first characterized TurboID and miniTurbo (Branon *et al.*, 2018). The main argument of these authors is that because TurboID is active without supplemental biotin, a "minus biotin" control in proteomics would still contain many biotinylated proteins, making it an ineffective negative control for identifying specific interactors. Therefore, other controls such as spatial controls are more effective in filtering out non-specific interactors.
4. We validated using imaging experiments that in HeLa cells expressing miniTurbo-YAP-EGFP, if we didn't add exogenous biotin, there is no background biotinylation signal, very different from when we added biotin (**reviewer Fig. 2.1 below**). This indicates that the background biotinylation is negligible, and our identified proteins are biotinylated through proximity to YAP.

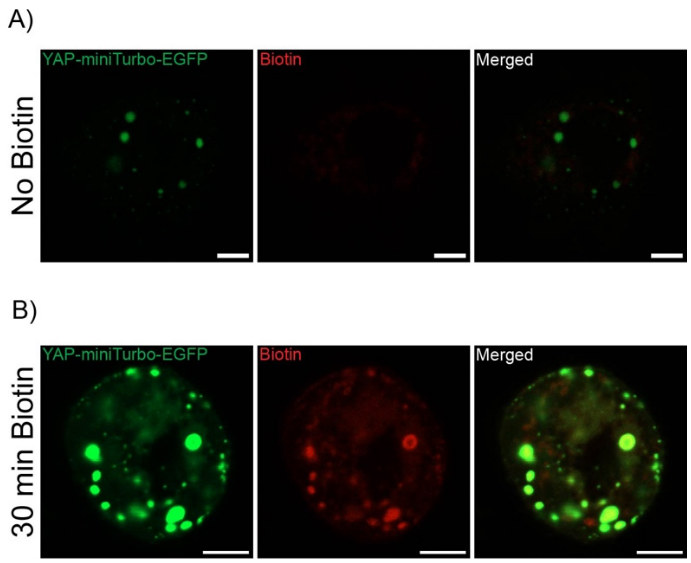

Reviewer Fig 2.1. Representative immunofluorescence images of HeLa cells transfected with YAP-miniTurbo-EGFP without (A) or with (B) 30 min incubation with biotin under 30 min of sorbitol treatment.

Small and Biologically Unexplained Proteome: The number of identified proteins (23 in control, 33 at 10 min, 39 at 2 h) 6 is strikingly low for proximity labeling. This limited output weakens confidence in the comprehensiveness of the dataset targeting a critical transcriptional co-activator.

We appreciate this comment by the reviewer. As demonstrated previously by Carr and Ting labs (Udeshi *et al.*, 2017), higher-specificity enrichment strategies—such as those focusing on direct biotinylation sites or using stringent washing—often yield smaller, higher-confidence datasets (dozens of hits) by filtering out indirect complex members and sticky background proteins. In this context, the 'missing' hits are often indirect interactors, and the 'clean' background reduces the dependence on statistical subtraction against a negative control. Indeed, our mass spectrometry data yielded high confidence candidates that are experimentally confirmed to be direct interactors of YAP in YAP condensates (**Figures 3-5**). Thank you again for the comment.

2. Contradictory Functional Requirement for Transcription. The core finding regarding function is inconsistent, severely questioning the requirement of TCF12/JUNB-dependent condensates for YAP activity: The depletion of TCF12 or JUNB abolishes condensate formation and decreases the expression of the YAP target gene Cyr61. However, the expression of another canonical YAP target gene CTGF, is reported as not significantly affected (ns) upon knockdown of either TCF12 or JUNB (Figure S3C, D). This is a major contradiction. If the TCF12/JUNB-dependent condensates are required for YAP transcriptional activation, then all downstream targets should be suppressed upon condensate abolition. The fact that CTGF remains unaffected suggests either that these condensates are dispensable for a subset of YAP function, or that the observed effect on Cyr61 is specific to a TCF12/JUNB regulatory mechanism independent of phase separation. This major inconsistency needs to be resolved through further functional data and a comprehensive discussion.

We appreciate the comment. Following the suggestion, we have now assayed additional Hippo pathway/ YAP target genes, including *BIRC5* and *AMOTL2* (**updated Figure 4H and I;**

reviewer Fig. 1.3). Our data shows that knocking down TCF12 and JUNB decreases the expression of these target genes, reflecting a broader impairment of YAP transcriptional activity. Considering our new data, we have added the following description to the main text: “Strikingly, TCF12 or JUNB knockdown impaired YAP transcriptional activity, reducing expression of the YAP target genes *CYR61* and *BIRC5*, and additionally lowering *AMOTL2* levels upon siTCF12 treatment”.

Our discovery that *CTGF* level did not change after siTCF12 or siJUNB (**Figure 4J**) indicated that *CTGF* expression may not be driven by YAP condensates in these U-2 OS cells. Indeed, YAP/TEAD can bind to both super enhancers (presumably where YAP condensates bind) and normal enhancers to drive downstream gene expression (Zanconato et al., 2018). We have now added the following description/discussion to the text: “The level of *CTGF*, however, is not affected after TCF12 or JUNB k.d., possibly because *CTGF* expression is mainly driven by YAP outside of condensates.” Thank you again for this very important question which helped advance the mechanistic insights of our paper.

3. Lack of Mechanistic and Biophysical Characterization. The manuscript provides no data to explain how TCF12 and JUNB influence phase separation. Missing Biophysical Data (FRAP): Phase separation is a biophysical process. Showing that TCF12/JUNB depletion prevents condensate formation (Figure 4) is purely correlative. The authors must perform Fluorescence Recovery After Photobleaching (FRAP) experiments to measure the exchange dynamics, mobility, and liquid-like properties of YAP condensates before and after knockdown. Do these proteins serve as scaffolds? Do they change the viscosity or maturation rate?

We appreciate the comments of the reviewer and agree that mechanistic insights into the scaffold/client relations among YAP, TCF12, and JUNB will be important. To this end, we performed FRAP on YAP condensates after TCF12/JUNB depletion using siRNA (**updated Figure 4K-M; reviewer Fig. 2.2 below**). We found that while the mobile fractions remained the same after siTCF12 and siJUNB, the $t_{1/2}$ of YAP condensates decreased after siJUNB, indicating that JUNB normally increases the stability/viscosity of YAP condensates. We have added the following description to the main text to describe our new data: “Lastly, JUNB also contributes to the stability of YAP condensates, as knocking it down increased the fluidity of the remaining YAP condensates (evidenced by a decrease of $t_{1/2}$ in fluorescence recovery after photobleaching experiments), while TCF12 k.d. did not have a similar effect.” Judging from our existing data that k.d. of TCF12 and JUNB both significantly decreased YAP condensate number without affecting YAP protein levels, we believe that TCF12 and JUNB are more likely to be scaffolds than passive clients, seeding YAP condensate formation. Thank you again for this question which prompted our new FRAP experiments, deepening the insights of our paper.

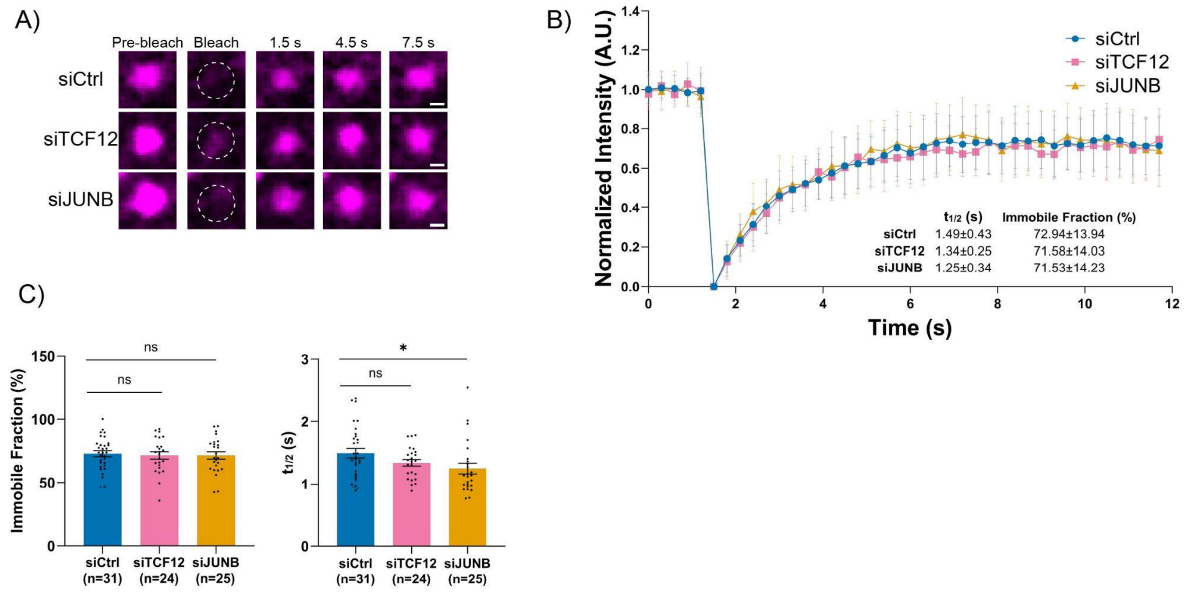

Reviewer Fig 2.2. JUNB regulated YAP condensate dynamics. (A) Representative imaging showing FRAP recovery of YAP-Halo condensate (labelled with JF646 Halo Dye) in siCtrl, siTCF12, and siJUNB cells, scale bars = 0.3 μm. (B) FRAP recovery curve (n=31 for siCtrl, n=24 for siTCF12, n=25 for JUNB). (C) Quantifications of immobile fraction (left) and t1/2 (right) from FRAP. Statistical significance was assessed using unpaired t-test; ns, not significant; * $p<0.05$. Data are shown as mean ± SEM.

4. Structural Basis and Scaffolding: There is no attempt to map the domains responsible for the interaction or nucleation. The manuscript claims TCF12 and JUNB are "required for YAP condensate formation", implying a scaffolding or nucleation role. The authors should investigate if they bind YAP directly and if they have unique low-complexity domains (IDRs) that facilitate multivalent interactions.

We appreciate the very important comment and suggestion of the reviewer. Following the advice from the reviewer, we have now mapped the domains in TCF12 and JUNB responsible for both direct YAP binding and incorporation into YAP condensates (**new Fig. 5; reviewer Fig. 1.1. above**). The results are interesting: while the DNA-binding (DB) domain of JUNB is required for both direct YAP binding and forming YAP condensates, the DNA-binding bHLH of TCF12 is required for direct YAP binding, and the IDR of TCF12 is required for incorporation into YAP condensates. We have added the following descriptions to the main text:

“To elucidate the molecular mechanisms by which TCF12 and JUNB regulate YAP condensate formation, we assessed a series of GFP-tagged domain deletion mutants for their interactions with endogenous YAP and their recruitments into condensates (Figure 5A). As expected, a GFP-only negative control failed to bind YAP or enrich within YAP condensates (Figure 5B, C, and E). TCF12 consists of an N-terminal intrinsically disordered region (IDR) and a C-terminal DNA-binding bHLH domain (Figure 5A). While wild-type GFP-TCF12 directly bound endogenous YAP and enriched at YAP condensates (Figure 5B, F, and I), deleting the IDR (ΔIDR) produced a decoupling effect: direct TCF12–YAP binding did not significantly change, yet condensate enrichment was significantly reduced (Figure 5B, D, G, and I). This indicates that while the IDR may be dispensable for direct YAP binding, it is essential for incorporation

into the condensate phase. Conversely, removing the bHLH domain (ΔbHLH) disrupted TCF12–YAP interactions and yet enhanced TCF12 recruitment and promoted the assembly of larger, more numerous YAP condensates (Figure 5B, D, H, and I; Supplemental Figure S4D and E). These results indicate that interestingly, TCF12 utilizes different domains for direct YAP binding (bHLH) and YAP condensate incorporation (IDR).

We performed a parallel analysis for JUNB, which contains two IDRs and a C-terminal DNA-binding (DB) domain (Figure 5A). In contrast to TCF12, JUNB's regulation of YAP appears primarily dependent on its DNA-binding capacity. Deletion of the DB domain (ΔDB) drastically reduced JUNB–YAP interactions and inhibited condensate formation (Figure 5C, D, K, and N; Supplemental Figure S4F and G). This suggests that YAP condensates nucleate near JUNB-bound genomic sites, and that the JUNBΔDB mutant acts as a dominant negative that prevents localized YAP concentration and condensation. On the other hand, neither IDR1 nor IDR2 was required for direct YAP binding, condensate incorporation, or the regulation of condensate morphology (Figure 5C, D, L, M, N; Supplemental Figure S4F and G). Taken together, these results indicate that the JUNB uses the DB for direct YAP binding and incorporation into YAP condensates."

We really appreciate the comments of the reviewer, which greatly improved the mechanistic insights of our paper.

5. Superficial Analysis of Proteomics Data Over Time. The analysis of the transition from 10 minutes to 2 hours post-sorbitol is descriptive and lacks biological interpretation:GO Term Interpretation (Figure 2E): The authors note the distinct clustering of proteins at 10 min and 2 hrs. The Gene Ontology (GO) term "Nucleic acid metabolic process" shows greater enrichment at 2 hours compared to 10 minutes (Figure 2E). Given the biological model-enhancer clustering (10 min) shifting to active transcription (2 h) -the authors need to discuss which specific nucleic acid metabolic proteins are enriched and propose a mechanism for their sequential recruitment in relation to the transcriptional activation phase. Simply presenting a bar plot of GO terms is insufficient for a research article.

Thank you for this important suggestion. To address this, we have investigated the specific proteins in each GO term, especially the "Nucleic acid metabolic process" at 2 hrs sorbitol treatment which is related to the gene expression process. We found that THRAP3 and ZMAT2 were especially interesting, and added the following description/discussion to the text: "Interestingly, more proteins were enriched after 2 hrs of sorbitol treatment during which YAP target genes have been activated (Cai *et al.*, 2019). Consistently, the Gene Ontology (GO) term "nucleic acid metabolic process" was highly enriched after 2 hrs of sorbitol treatment, and included unique proteins such as THRAP3 (thyroid hormone receptor-associated protein 3, a transcriptional coactivator) and ZMAT2 (zinc finger matrin-type 2, a component of the spliceosome) (Figure 2E; Supplemental Figure S2), indicating that YAP condensates may engage with transcriptional cofactors and the splicing machinery to accelerate downstream gene expression."

We cannot speculate how these unique proteins start interacting with YAP at 2 hrs of sorbitol treatment. Therefore, we added the following sentence: "While outside the scope of the current study, future studies will test how these factors may drive gene expression for surviving hyperosmotic stress." Thank you again for the valuable advice.

Minor Points

1. Data Presentation and Visualization, Error Bar Clarity: The legend for Figure 4 states that error bars represent mean SD, while the legend for Figure 1 and others states mean SEM. This discrepancy must be corrected for statistical consistency throughout the paper.

We apologize for the oversight: the error bars should be SEM. We have corrected the figure legends. Thank you again for the comments.

YAP Quantification Context: In Figure 4G, the Western blot and subsequent quantification show the effect of TCF12/JUNB knockdown on both YAP-Halo and endogenous YAP. Please ensure the quantification clearly specifies how the signals from the exogenous YAP-HaloTag fusion protein and the endogenous wild-type YAP protein are integrated or separated, particularly since the siRNA targets the endogenous system.

We have now specified in the figure legend that the YAP expression combines both tagged and untagged endogenous YAP. Thank you for this suggestion which improved the clarity of our paper.

Localization of IFI16 (Figure 3G): IFI16 appears to be concentrated heavily around the nuclear periphery in the "No Sorbitol" condition. This known shuttling or peripheral localization should be noted in the text, as it adds context to its subsequent enrichment inside the nuclear condensates upon sorbitol treatment.

This is a great observation. We have now included the following description in the main text: “Among the candidates, while TCF12 (transcription factor 12) and JUNB (transcription factor JunB) remained nuclear both before and after sorbitol treatment, IFI16 (interferon alpha-inducible protein 6) underwent a striking relocalization after sorbitol treatment, shifting from predominantly cytoplasmic and peri-nuclear to nuclear-localized (Figs. 3C-H).” Thanks again for the suggestion.

2. Technical and Terminological Clarity Missing Primer Information: While primers for CTGF are listed in the Materials and Methods, the primers used for Cyr61 (quantified in Figure 4H, I) should also be provided for full reproducibility. Clarity on IFI16 Necessity: The authors state they could not test the necessity of IFI16 because 3 days of knockdown affected cell viability. This should be stated clearly as an experimental limitation in the main text.

We have now provided the primer sequences for all the target genes we tested in the Methods Section. Following the suggestion of the reviewer, we have added a section titled Limitations of the Study, and clearly noted such limitations as: “Our IFI16 k.d. experiments killed the cells so we cannot access the roles of IFI16 in YAP condensation. The interaction studies of YAP with TCF12, JUNB, and their respective mutants were done in the presence of endogenous TCF12 and JUNB.” Thank you for the suggestion.

Protein Exclusions: The authors should briefly clarify the selection criteria used to narrow the list of 33 identified proteins at 10 min (Figure 2A) down to the handful of candidates selected for IF validation (Figures 3 and S2).

We chose the proteins of interest based on their presence at 10 minutes, nuclear expression, functions related to transcription and RNA splicing, and the availability of high quality, commercially available antibodies against them. Structural features such as coiled-coiled domain and disordered region content were considered but ultimately not used as dominant factors for narrowing down the list. We added the following sentence to the paper to briefly

explain our selection criteria: “Among the 33 candidate proteins enriched in the 10 min sorbitol treated sample, 8 candidates have transcription and RNA splicing related activities”.

References for the Rebuttal:

Branon, T.C., Bosch, J.A., Sanchez, A.D., Udeshi, N.D., Svinkina, T., Carr, S.A., Feldman, J.L., Perrimon, N., and Ting, A.Y. (2018). Efficient proximity labeling in living cells and organisms with TurboID. Nat Biotechnol *36*, 880-887.
Cai, D., Feliciano, D., Dong, P., Flores, E., Gruebele, M., Porat-Shliom, N., Sukenik, S., Liu, Z., and Lippincott-Schwartz, J. (2019). Phase separation of YAP reorganizes genome topology for long-term YAP target gene expression. Nat Cell Biol *21*, 1578-1589.
Larochelle, M., Bergeron, D., Arcand, B., and Bachand, F. (2019). Proximity-dependent biotinylation mediated by TurboID to identify protein–protein interaction networks in yeast. Journal of Cell Science *132*.
Su, Y., Guo, Y., Guo, J., Zeng, T., Wang, T., and Liu, W. (2023). Study of FOXO1-interacting proteins using TurboID-based proximity labeling technology. BMC Genomics *24*, 146.
Udeshi, N.D., Pedram, K., Svinkina, T., Fereshetian, S., Myers, S.A., Aygun, O., Krug, K., Clauser, K., Ryan, D., Ast, T., Mootha, V.K., Ting, A.Y., and Carr, S.A. (2017). Antibodies to biotin enable large-scale detection of biotinylation sites on proteins. Nat Methods *14*, 1167-1170.
Zhang, Y., Song, G., Lal, N.K., Nagalakshmi, U., Li, Y., Zheng, W., Huang, P.-j., Branon, T.C., Ting, A.Y., Walley, J.W., and Dinesh-Kumar, S.P. (2019). TurboID-based proximity labeling reveals that UBR7 is a regulator of N NLR immune receptor-mediated immunity. Nature Communications *10*, 3252.

RE: Manuscript #E25-11-0532R
TITLE: "Investigating the functional components of YAP condensates"

Dear Dr. Cai:

I am pleased to accept your manuscript for publication in Molecular Biology of the Cell.

Sincerely,
Huaiying Zhang
Monitoring Editor
Molecular Biology of the Cell

Dear Dr. Cai:

Congratulations on the acceptance of your manuscript! Thank you for publishing your work in Molecular Biology of the Cell (MBoC).

Within 10 days, an unedited PDF of your manuscript will be published on MBoC in Press, an early-release journal version. The date your manuscript appears on this site is the official publication date.

Your copyedited and typeset manuscript will be scheduled for publication in the next available issue of MBoC. Once your paper is ready for review, our production team will notify you. In the summer of 2024, our production provider will introduce an online proofing solution, which will streamline the process of checking and validating author changes. This will result in improved typesetting quality, enhanced digital content, and a faster overall process.

We invite you to submit images related to your accepted manuscript to be considered for the journal cover.
Cover images must contain color, TIF Format
Size image at 7.4" wide x 5.85" deep
RGB and CMYK color (please submit two separate files, one in each format)
300 dpi resolution

Please also send a 1-paragraph description of the image that a general audience could understand. Include with the caption the name and institution of the individual(s) the image should be attributed to. Please provide this description in a Word file. You can send the file via email or by your favorite file transfer service to mboc@ascb.org.

Authors of Articles and Brief Communications are also encouraged to create a short video abstract to accompany their article when it is published. These video abstracts, known as Science Sketches, are up to 2 minutes long and will be published on YouTube and then embedded in the article abstract. Science Sketch Editors on the MBoC Editorial Board will guide you as you prepare your video. Information about how to prepare and submit a video abstract is available at www.molbiolcell.org/science-sketches. Please contact mboc@ascb.org if you are interested in creating a Science Sketch.

We look forward to publishing your paper in MBoC.
Sign up for MBoC's new content alerts here https://tinyurl.com/3bcsyk3e

Sincerely,

Eric Baker
Journal Production Manager
MBoC Editorial Office
mbc@ascb.org

Reviewer #1 (Remarks to the Author):

This revision has already addressed all my concerns. It has been greatly improved. Good to be published.
